# Corneal strain influences keratocyte proliferation and migration through upregulation of ALDH3A1 expression

**DOI:** 10.1096/fj.202401392R

**Published:** 2024-12-09

**Authors:** Qian Zhang, Xin Zhou, Wei Zhang, Xiaolei Wang, Shengqian Dou, Leilei Zhao, Roine El‐Habta, Qingjun Zhou, Ludvig J. Backman, Patrik Danielson

**Affiliations:** ^1^ Department of Medical and Translational Biology Umeå University Umeå Sweden; ^2^ School of Medicine Southeast University Nanjing China; ^3^ State Key Laboratory Cultivation Base, Shandong Provincial Key Laboratory of Ophthalmology Eye Institute of Shandong First Medical University Qingdao China; ^4^ Medical College Qingdao University Qingdao China; ^5^ Department of Community Medicine and Rehabilitation, Section of Physiotherapy Umeå University Umeå Sweden; ^6^ Department of Clinical Sciences, Ophthalmology Umeå University Umeå Sweden

**Keywords:** ALDH3A1, biomechanics, corneal injuries, corneal strain, keratocytes, migration, NF‐κB, proliferation

## Abstract

Keratocytes are the primary resident cells in the corneal stroma. They play an essential role in maintaining corneal physiological function. Studying the factors that affect the phenotype and behavior of keratocytes offers meaningful perspectives for improving the understanding and treatment of corneal injuries. In this study, 3% strain was applied to human keratocytes using the Flexcell® Tension Systems. Real‐time quantitative PCR (RT‐qPCR) and western blot were used to investigate the influence of strain on the expression of intracellular aldehyde dehydrogenase 3A1 (ALDH3A1). ALDH3A1 knockdown was achieved using double‐stranded RNA‐mediated interference (RNAi). Immunofluorescence (IF) staining was employed to observe the impact of changes in ALDH3A1 expression on nuclear factor kappa‐light‐chain‐enhancer of activated B cells (NF‐κB) nuclear translocation. Keratocyte proliferation and migration were assessed by bromodeoxyuridine (BrdU) assay and scratch wound healing assay, respectively. Mouse injury models and single‐cell RNA sequencing of keratocytes from keratoconus patients were used to assess how strain influenced ALDH3A1 in vivo. Our results demonstrate that 3% strain suppresses keratocyte proliferation and increases ALDH3A1. Increased ALDH3A1 inhibits NF‐κB nuclear translocation, a key step in the activation of the NF‐κB signaling pathway. Conversely, ALDH3A1 knockdown promotes NF‐κB nuclear translocation, ultimately enhancing keratocyte proliferation and migration. Elevated ALDH3A1 levels were also observed in mouse injury models with increased corneal strain and keratoconus patients. These findings provide valuable insights for further research into the role of corneal strain and its connection to corneal injury repair.

## Graphical abstract

A significant factor contributing to scar formation after corneal injury is the abnormal proliferation and migration of keratocytes to the wounded area. Corneal strain inhibits NF‐κB nuclear translocation, a critical step in activating the NF‐κB signaling pathway, through the upregulation of ALDH3A1 expression. This process ultimately suppresses keratocyte proliferation and migration. Our findings provide valuable insights for further research into the role of corneal strain and its correlation with the repair of corneal injuries.

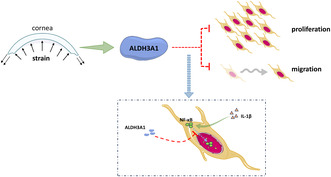

**Abbreviations**
ALDH3A1: aldehyde dehydrogenase 3A1
BrdU: bromodeoxyuridine
DMEM/F‐12: Dulbecco's Modified Eagle Medium:Nutrient Mixture F‐12
ECM: extracellular matrix
EdU: 5 ethynyl‐2'‐deoxyuridine
FBS: Fetal Bovine Serum
HBSS: Hanks' Balanced Salt Solution
IF: Immunofluorescence
IL‐1β: interleukin‐1 beta
IL‐8: interleukin‐8
IOP: intraocular pressure
NF‐κB: nuclear factor kappa‐light‐chain‐enhancer of activated B cells
NT siRNA: non‐targeting small interfering RNA
RNAi: Double‐stranded RNA‐mediated interference
RT‐qPCR: Real‐time quantitative PCR
scRNA‐Seq: Single‐cell RNA sequencing
siRNA: small interfering RNA
TGF‐β: transforming growth factor‐β
TPCA‐1: 2‐[(aminocarbonyl)amino]‐5 ‐(4‐fluorophenyl)‐3‐thiophenecarboxamide
UV: ultraviolet

## 1 INTRODUCTION

Corneal injuries often result in impaired vision or even blindness. Keratocytes, as the primary resident cells in the corneal stroma, play a crucial role in preserving normal corneal physiological function and facilitating the repair of corneal injuries.^1^, ^2^ Exploring the factors influencing the phenotype and cell behavior of keratocytes provides valuable insights for enhancing the comprehension and treatment of corneal injuries.

The cornea is a viscoelastic, transparent, and highly organized tissue that forms the outermost layer of the human eye.^3^, ^4^ The human corneal stroma is a fibrous, tough, and transparent layer located between the outer epithelium and the inner endothelium of the cornea. The stroma constitutes approximately 90% of the cornea's overall thickness.^5^ Keratocytes, the dominant cells in the corneal stroma, are dendritic mesenchymal cells scattered throughout this layer.^2^, ^6^ Under normal physiological conditions, keratocytes remain in a quiescent state with minimal proliferation or migration.^1^, ^7^ Their primary function is to maintain the stromal structure and significantly contribute to corneal transparency and homeostasis by synthesizing and secreting various extracellular matrix (ECM) components.^8^ During corneal injury or disease, quiescent keratocytes are activated by various cytokines, including interleukin‐1 beta (IL‐1β).^9^ The activated keratocytes undergo phenotypic transformation to fibroblasts and subsequently myofibroblasts that initiate cell proliferation, migration, and accelerate collagen production.^10^, ^11^ This transformation is primarily triggered and governed by the interplay between IL‐1β and the transforming growth factor‐β (TGF‐β) system.^9^ Furthermore, the mechanism underlying this process is controlled by the interplay of nuclear factor kappa‐light‐chain‐enhancer of activated B cells (NF‐κB) signaling.^9^ It is well‐established that keratocytes exert a pivotal influence in mediating the corneal response to injuries.^8^, ^12^, ^13^

The strain of a cornea refers to the deformation experienced by the corneal tissue.^14^, ^15^, ^16^ In vivo, a healthy cornea always has a certain strain, which is mainly caused by intraocular pressure (IOP) and the pressure from the external environment.^3^ The dome‐shape and mechanical properties of the cornea are essential for maintaining its normal physiological characteristics.^5^, ^17^, ^18^ It is reported that the natural expansional elasticity deformation of the cornea commonly varies between 0% and 3.5%.^19^ Corneal diseases or injuries typically alter the strain of the cornea, ultimately affecting its physiological properties, leading to visual damage.^10^, ^20^, ^21^, ^22^, ^23^

Considerable efforts have been made to understand the factors influencing keratocyte phenotype and behavior, highlighting the importance of corneal biomechanics. Currently, few studies focus on how corneal strain regulates keratocyte phenotype and behavior. However, many studies recognize that corneal biomechanics significantly affect keratocytes. A study demonstrated that keratocytes derived from human‐induced pluripotent stem cells exhibit significantly varying levels of keratocyte markers, such as aldehyde dehydrogenase 3A1 (ALDH3A1) and LUMICAN, and display distinctly different morphologies, based on the stiffness of the substrate they are cultured on.^24^ There are also relevant findings on the impact of substrate stiffness on keratocyte behaviors such as cell adhesion, proliferation, migration, and differentiation.^25^, ^26^ Moreover, the responses of keratocytes to specific wound healing cytokines can be modulated by the biomechanical properties of the corneal stromal ECM.^27^ A previous study from our group has shown that 3% dome‐shaped static mechanical strain (referring to 3% strain in the text below) upregulates the expression of the keratocyte biomarkers LUMICAN and KERATOCAN, and contributes to maintaining keratocyte phenotype.^17^, ^28^

ALDH3A1 is an abundantly expressed protein in the cornea of mammals, accounting for 5%–50% of the total water‐soluble protein fraction, depending on the species.^29^ Research indicates that ALDH3A1 plays a crucial role in maintaining corneal transparency^30^, ^31^ and aids in the defense against ultraviolet (UV)‐induced oxidative stress.^32^, ^33^ Recent studies indicate that ALDH3A1 may also function as a regulator of the cell cycle,^34^ with either promotional^35^, ^36^, ^37^, ^38^ or inhibitory^33^, ^34^, ^39^, ^40^ effects on cell proliferation, depending on cell type. ALDH3A1 has also been reported to be associated with cell migration,^36^, ^38^, ^41^ and its expression shows potential in enhancing wound healing.^33^ Until now, there have been few studies investigating the correlation between corneal strain and ALDH3A1.

Our previously published findings demonstrate that in keratocytes, the activation of the NF‐κB signaling pathway by IL‐1β leads to a decrease in the mRNA level of *ALDH3A1*. Conversely, in the presence of the NF‐κB signaling pathway inhibitor 2‐[(aminocarbonyl)amino]‐5‐(4‐fluorophenyl)‐3‐thiophenecarboxamide (TPCA‐1), the IL‐1β‐induced effect on NF‐κB is reversed, resulting in a restoration of ALDH3A1 expression levels.^9^

In this study, we exposed human keratocytes to 3% dome‐shaped mechanical strain to explore its impact on the expression of ALDH3A1 and its subsequent effects on keratocyte proliferation and migration. Our results reveal that the exposure of 3% strain upregulates the expression of ALDH3A1 in keratocytes. This upregulation of ALDH3A1 inhibits NF‐κB nuclear translocation, a crucial step for the activation of NF‐κB signaling pathway. However, when ALDH3A1 expression is knocked down, NF‐κB nuclear translocation is promoted, ultimately resulting in increased keratocyte proliferation and migration. To our knowledge, this is the first demonstration of the influence of mechanical strain on keratocyte ALDH3A1 expression and its impact on the process of keratocyte proliferation and migration. Our findings offer valuable insights for further research into corneal strain and its correlation with the repair of corneal injuries.

## 2 MATERIALS AND METHODS

### 2.1 Human cornea samples collection

Healthy human corneas were provided by the Tissue Establishment, Eye Bank Umeå, at the University Hospital of Umeå, Sweden. The corneas were donated voluntarily by individuals who chose to donate their corneas post‐mortem during their lifetime for both transplantation and research purposes. In cases where the corneas were utilized for transplantation, the leftover part of the cornea was delivered to the laboratory for research. The study underwent review by the Regional Ethical Review Board in Umeå, which determined it to be exempt from the requirement for approval (2010‐373‐31M).

All the cornea samples collected for research purposes were anonymized, only labeled with donors' sex and age (see Table 1). This study was conducted in accordance with the principles of the Declaration of Helsinki.

**TABLE 1:** Donor information for cornea samples.

Sample ID	Sex	Age
B280	Male	72
B281	Male	72
B286	Male	82
B288	Female	72
B302	Male	72
B309	Male	26
B314	Male	65
B315	Female	65

### 2.2 Human primary keratocytes isolation and culture

Isolation and culture methods of human primary keratocytes were described in detail previously.^42^ In brief, the human cornea sample was washed twice in Hanks' Balanced Salt Solution (HBSS) (Gibco, 14 170‐088, Life Technologies, Scotland, UK), before and after the epithelium and endothelium of the cornea were gently removed by scraping with a sterile scalpel. The remaining part of the cornea is the corneal stroma. The corneal stroma was cut into small pieces and transferred into a 15 mL sterile centrifuge tube with 2 mL Dulbecco's Modified Eagle Medium:Nutrient Mixture F‐12 (DMEM/F‐12) (Gibco, 31330095, Life Technologies, Scotland, UK) containing 2% Fetal Bovine Serum (FBS) (Sigma‐Aldrich, F9665, St. Louis, MO, USA) and 2 mg/mL Collagenase from Clostridium histolyticum (Sigma‐Aldrich, C0130, St. Louis, MO, USA). The corneal stroma was incubated at 37°C in a humidified incubator with 5% CO_2_ overnight. Subsequently, the homogenized sample was centrifuged at 200 *g* for 5 min at room temperature before the supernatant was discarded. The resulting cell pellet, containing keratocytes, was resuspended with DMEM/F‐12 containing 2% FBS and seeded into a 25 cm^2^ flask. The culture medium was renewed every second day. Confluent keratocytes were passaged at a ratio of 1:3. The cultured cells were characterized across all experimental conditions, confirming the presence of an unchanged keratocyte phenotype (see Figure S1 and Table S1), as previously described by us.^42^

### 2.3 Animals

Male C57BL/6J mice, aged 8 weeks, were purchased from Beijing Vital River Laboratory Animal Technology Co., Ltd. (Beijing, China). The experimental procedures adhered to the ARVO Statement for the Use of Animals in Ophthalmic and Vision Research and were approved by the Ethical Committee of Shandong Eye Institute (Approval No: 2020‐G‐08).

### 2.4 Mechanical loading

Keratocytes were seeded at a density of 1.5 × 10^5^ cells per well in the Bioflex 6‐well plate (Flexcell, BF‐3001C, Burlington, NC, USA) and incubated at 37°C in a humidified incubator with 5% CO_2_. The next day, the Bioflex 6‐well plate was placed on the BioFlex® Loading Stations™ (Flexcell, LS‐3000B25.VJW, Burlington, NC, USA) with dome‐shaped loading post of 25 mm diameter. This mimicked the shape of a healthy human cornea in native circumstances (1:2 ratio). The keratocytes were then kept under 3% equibiaxial strain consistently for 48 h. Keratocytes cultured in the same condition but without being exposed to strain served as the control group.

### 2.5 Double‐stranded RNA‐mediated interference (RNAi)

Keratocytes were seeded at a density of 1.5 × 10^5^ cells per well in the 6‐well plate and incubated at 37°C in a humidified incubator with 5% CO_2_ overnight. For each well to be transfected, RNAi duplex‐Lipofectamine™ RNAiMAX complexes were prepared as follows: (a) 25 pmol ALDH3A1 siRNA (Invitrogen, siRNA ID: s1243, Carlsbad, California, USA) was diluted in 125 μL Opti‐® I Reduced Serum Medium (Gibco, 31985070, Life Technologies Corporation, Grand Island, NY, USA) without serum. (b) 6 μL of Lipofectamine™ RNAiMAX Transfection Reagent (Invitrogen, 13778075, Carlsbad, CA, USA) was diluted in 125 μL Opti‐MEM® I Reduced Serum Medium. (c) The RNAi duplex (a) was combined with the diluted Lipofectamine™ RNAiMAX (b) and incubated for 5 min at room temperature. Finally, the RNAi duplex‐Lipofectamine™ RNAiMAX complexes (250 μL per well) were added to each well containing cells and culture media without antibiotics. Keratocytes were incubated for 48 h at 37°C in a humidified incubator with 5% CO_2_. Silencer™ Select Negative Control No. 1 siRNA (Invitrogen, 4390843, Carlsbad, CA, USA) was used as negative control siRNA. When transfection was conducted in the 24‐well plate, the transfection was initiated by supplementing 5 pmol of siRNA and 1.2 μL of Lipofectamine™ RNAiMAX Transfection Reagent, diluted in 50 μL Opti‐MEM® I Reduced Serum Medium. When transfection was conducted in the 96‐well plate, the transfection was initiated by supplementing 1 pmol of siRNA and 0.2 μL of Lipofectamine™ RNAiMAX Transfection Reagent, diluted in 10 μL Opti‐MEM® I Reduced Serum Medium.

### 2.6 RNA extraction and cDNA synthesis

Total RNA of keratocytes were extracted according to the user manual of RNeasy Mini Kit (Qiagen, 74104, Hilden, North Rhine Westphalia, Germany). Subsequently, total RNA of keratocytes were reverse transcribed into cDNA according to the manufacturer's protocol of High‐Capacity cDNA Reverse Transcription Kit (Applied Biosystems™, 4368814, Vilnius, Lithuania).

### 2.7 Real‐time quantitative PCR (RT‐qPCR) assay

RT‐qPCR assay was performed according to the user manual of TaqMan™ Fast Advanced Master Mix (Applied Biosystems™, 4444963, CA, USA). Probes of target genes for RT‐qPCR assay were *ALDH3A1* probe (ThermoFisher; Hs00964880_m1) and *IL‐8* probe (ThermoFisher; Hs00153133_m1). The probe of the reference gene was *GAPDH* probe (ThermoFisher; Hs02786624_g1). The amplification was performed using the ViiA7 Real‐Time PCR system (Applied Biosystems™, Carlsbad, CA, USA), and the reaction condition includes an initial step of 20 s at 95°C, followed by 40 cycles of a 1 s denaturation at 95°C and then 20 s annealing and extension at 60°C. Results were analyzed using the 2^−∆∆Ct^ method.

### 2.8 Cell lysis protein extraction

Plates seeded with keratocytes were placed on ice and washed with ice‐cold PBS (Medicago, 09‐8912‐100, Vantaa, Finland). Ice‐cold RIPA (Thermo Scientific, 89901, Rockford, IL, USA) with Halt™ Protease and Phosphatase Inhibitor Single‐Use Cocktail (100X) (Thermo Scientific, 78446, Rockford, lL, USA) was added to the cells and scraped off the plate using a cold plastic cell scraper. The cell suspension was transferred into a pre‐cooled 1.5 mL tube and constantly agitated for 30 min at 4°C. The cell suspension was centrifuged at 16 000 *g* for 20 min at 4°C before the supernatant was collected to a fresh pre‐cooled 1.5 mL tube. Cell lysis protein was stored at −80°C.

### 2.9 Cytoplasmic and nuclear protein extraction

Cytoplasmic and nuclear protein extraction was performed according to the manufacturer's protocol of NE‐PER Nuclear and Cytoplasmic Extraction Reagents (Thermo Scientific, 78833, Rockford, IL, USA). Briefly, adherent cells were harvested with 0.05% Trypsin–EDTA (Gibco, 25300054, Grand Island, NY and Scotland, UK). Cell suspension was centrifuged at 500 *g* for 5 min at 4°C before washed in ice‐cold PBS and centrifuged again at 500 *g* for 3 min at 4°C. Ice cold CER I with Halt™ Protease and Phosphatase Inhibitor Single‐Use Cocktail (100X) was added to the cell pellet and vigorously vortexed for 15 s before incubated on ice for 10 min and then centrifuged again. Ice cold CER II was added to the cell pellet and vortexed for 5 s followed by incubated on ice for 1 min and vortexed for 5 s. The suspension was centrifuged at 16 000 *g* for 5 min at 4°C. The supernatant (cytoplasmic extract, recorded as CPE) was transferred to a pre‐cooled clean 1.5 mL tube. The insoluble fraction was resuspended in ice‐cold NER buffer with Halt™ Protease and Phosphatase Inhibitor Single‐Use Cocktail (100X) and vortexed for 15 s. The sample was incubated on ice and vortexed for 15 s every 10 min for 40 min in total. The suspension was centrifuged at 16 000 *g* for 10 min at 4°C and the supernatant (nuclear extract, recorded as NE) was transferred to a pre‐cooled clean 1.5 mL tube. Cytoplasmic and nuclear protein extractions were stored at −80°C.

### 2.10 Western blot

The concentration of protein samples was determined according to the user manual of Pierce™ BCA Protein Assay Kit (Thermo Scientific, 23227, Rockford, IL, USA). 10 μg of protein of each sample was heat‐denatured and loaded onto Mini‐PROTEAN® TGX™ Precast Protein Gels (Biorad, 4561044, 4561096, Hercules, CA, USA). Proteins were separated by gel electrophoresis for 1 h at 110 V and then transferred onto a PVDF Transfer Membranes (Thermo Scientific, 88520, Rockford, IL, USA) for 1 h at 100 V. Membranes were blocked with 5% BSA (Sigma‐Aldrich, A9647, St. Louis, MO, USA) in TBS (Biorad, 1706435, Hercules, CA, USA) with 0.1% Tween 20 (VWR, 9005‐64‐5, France) for 30 min at room temperature, followed by incubation with appropriate primary antibody (primary antibodies involved in the western blot experiment are listed below: Anti‐ALDH3A1 antibody (Abcam, ab129022, Waltham, Boston, USA), NF‐κB p65 (D14E12) XP® Rabbit mAb (Cell signaling, 8242, Danvers, Massachusetts, USA), β‐Actin Antibody (Cell signaling, 4967, Danvers, Massachusetts, USA), Histone H3 Antibody (Cell signaling, 9715, Danvers, Massachusetts, USA), Recombinant Anti‐alpha Tubulin antibody [EP1332Y] (Abcam, ab52866, Waltham, Boston, USA)) overnight at 4°C. Membranes were washed with TBST and incubated with Anti‐rabbit IgG, HRP‐linked Antibody (Cell signaling, 7074, Danvers, Massachusetts, USA) for 1 h at room temperature. Membranes were subsequently washed with TBST and incubated with SuperSignal™ West Pico PLUS Chemiluminescent Substrate (Thermo Scientific, 34577, Carlsbad, CA, USA) for 10 s and analyzed using an Odyssey Fc Imaging System (LI‐COR Biosciences, Lincoln, Nebraska, USA). Densitometry of the bands was quantified using ImageJ software (version 1.53, National Institutes of Health, USA).

### 2.11 Measurement of proliferation rate

To assess the proliferation rate of keratocytes using the colorimetric BrdU assay, 5 × 10^3^ keratocytes were seeded per well in a 96‐well plate and left at 37°C in a humidified incubator with 5% CO_2_ for 24 h to fully recover. To assess the influence of ALDH3A1 on the cell proliferation, the cells were transfected with *ALDH3A1* siRNA or Silencer™ Select Negative Control No. 1 siRNA for 48 h, as previously described. To assess the influence of IL‐1β on the cell proliferation, the cells were exposed to 1 ng/mL Human IL‐1 beta Recombinant Protein (Gibco, PHC0815, Frederick, MD, USA)^9^ for 0, 12, or 24 h, respectively. The cell proliferation rate was measured according to the user manual of the Cell Proliferation ELISA, BrdU (colorimetric) kit (Roche, 11647229001, Mannheim, Germany). In brief, BrdU labeling solution was added to the culture wells to a final concentration of 10 μmol/L and incubated for 2 h at 37°C. The cells were fixed for 30 min at room temperature and treated with anti‐BrdU‐peroxidase working solution for 90 min. The antibody conjugate was then removed, and the cells were washed three times in PBS. For color development, substrate solution was added for 20 min. The absorbance was measured using a spectrophotometer set at 370 nm (reference wavelength 492 nm).

To evaluate the proliferation rate of keratocytes using the EdU staining assay, 1.5 × 10^5^ keratocytes were seeded per well in a Bioflex 6‐well plate and incubated at 37°C in a humidified incubator with 5% CO₂. The following day, the cells were exposed to 3% static mechanical strain, as previously described, for a total of 48 h. At the 24‐h point of strain application, 1 ng/mL IL‐1β (Gibco, PHC0815, Frederick, MD, USA) was added to selected groups. The proliferation rate was measured according to the user manual of the Click‐iT® Plus EdU Imaging Kit (Invitrogen, C10640, Carlsbad, CA, USA). Briefly, at the 24‐h point, EdU was added to the cell medium at a final concentration of 10 μM, and cells continued to be strained for an additional 24 h. Afterward, cells were fixed in 3.7% formaldehyde in PBS at room temperature for 15 min and permeabilized in 0.5% Triton® X‐100 in PBS for 20 min at room temperature. The samples were then treated with the Click‐iT® Plus reaction cocktail for 30 min at room temperature. Subsequently, the cells were stained with Hoechst® 33342 for 30 min at room temperature and imaged using a Leica Thunder Widefield microscope (Leica Microsystems, Wetzlar, Germany) at 200× magnification. For each group, nine randomly selected fields of view were imaged. ImageJ (version 1.53, National Institutes of Health, USA) was used to count the EdU‐ and Hoechst 33342‐positive cells. The proliferation rate was calculated as the percentage of EdU‐positive cells among the total Hoechst 33342‐positive cells. The mean proliferation rate across the 9 fields of view for each group was then used for statistical analysis.

### 2.12 Scratch wound healing assay

0.35 × 10^5^ keratocytes were seeded per well in a 24‐well plate and left at 37°C in a humidified incubator with 5% CO_2_ for 24 h to fully recover.

To assess the influence of IL‐1β on the cell migration, a scratch was made along the entire length of each well with a 1 mL pipette tip before 1 ng/mL IL‐1β was administrated. To assess the influence of ALDH3A1 on the cell migration, the cells were transfected with ALDH3A1 siRNA or Silencer™ Select Negative Control No. 1 siRNA for 48 h, as described previously. A scratch was made along the entire length of each well with a 1 mL pipette tip after 48 h of siRNA transfection.

The plates were then placed in an IncuCyte Live‐Cell Analysis System (Sartorius, Göttingen, Germany), and photographs were taken every 12 h for 48 h. A scratch with a length of 436 mm was randomly selected from each well. The remaining wound area of the selected scratch was measured using ImageJ (version 1.53, National Institutes of Health, USA) and a plugin for the quantification of the wound area.^43^

### 2.13 Cornea mechanical injury model

The mouse corneal mechanical injury model was established based on the previous description with appropriate modifications.^44^, ^45^ In brief, mice were anesthetized through intraperitoneal injection of 0.6% pentobarbital sodium, followed by the topical application of 2% xylocaine. In the scratch injury model, the mouse central corneal epithelium (3 mm diameter) and anterior stromal portion were removed using an AlgerBrush II Rust Ring Remover (Alger, 3943051, Lago Vista, TX, USA). In the cut injury model, the mouse central corneal epithelium (3 mm diameter) was removed using an AlgerBrush II Rust Ring Remover, followed by cutting anterior stromal portion with curved corneal scissors (Mingren eye instruments, Suzhou, China). The injury extends to approximately one‐third of the stroma thickness in both models. In both models, ofloxacin eye drops (Santen Pharmaceutical (China) Co., Ltd., Suzhou, Jiangsu, China) were administered to prevent infection. A single eye was utilized in each mouse for all experiments. The intraocular pressure (IOP) was measured before the injury induction and at 7 days post‐injury.

### 2.14 Mouse corneal thickness measurement

Mouse corneal thickness measurements were performed at six randomly selected sites within images of central corneal cryosections using ImageJ software (version 1.53, National Institutes of Health, USA).

### 2.15 IOP measurement

Following anesthesia, IOP was assessed using a TonoLab rebound tonometer (iCare, Vantaa, Finland). This device automatically derived the mean value from six measurements after excluding the highest and lowest values. This automatically machine‐generated mean result was treated as one reading, and five readings were acquired from each eye. The means of these five readings were calculated to determine the overall mean IOP.

### 2.16 Immunofluorescence

Cells cultured in the Bioflex 6‐well plate were fixed with formalin solution, neutral buffered, 10% (approx. 4% formaldehyde) (Sigma‐Aldrich, HT501128, St. Louis, MO, USA) for 20 min and then permeabilized with 0.15% Triton X‐100 (Sigma‐Aldrich, 9036‐19‐5, St. Louis, MO, USA) in PBS for 10 min at room temperature. Then the cells were incubated with the primary antibody against NF‐κB p65 (Cell Signaling, 8242, Danvers, Massachusetts, USA) for 1 h after the incubation with 5% BSA in PBS for 30 min at room temperature. Cells were washed with PBS and incubated with Goat anti‐Rabbit IgG (H + L) Highly Cross‐Adsorbed Secondary Antibody, Alexa Fluor™ Plus 594 (Invitrogen, A32740, Carlsbad, CA, USA) for 1 h at room temperature. Thereafter, cells were stained with DAPI (Thermo Scientific, 62248, Rockford, lL, USA) for 15 min at room temperature and imaged using Leica Thunder Widefield microscope (Leica Microsystems, Wetzlar, Germany) with 400‐fold magnification.

Mouse corneal cryosections were fixed in paraformaldehyde, 4% in PBS (Thermo Scientific Chemicals, J61899.AP, Waltham, Massachusetts, USA) for 10 min at room temperature. Following permeabilization with 0.2% Triton X‐100 in PBS, samples were blocked with 2% BSA and then incubated with the primary antibodies against ALDH3A1 (Abcam, ab76976, Waltham, Boston, USA) overnight at 4°C. Subsequently, sections were washed and incubated with Donkey anti‐Rabbit IgG (H + L) Highly Cross‐Adsorbed Secondary Antibody, Alexa Fluor™ 488 (Invitrogen, A‐21206, Carlsbad, CA, USA) for 1.5 h followed by DAPI for 15 min at room temperature. Samples were washed with PBS and imaged using ECHO Revolve microscope (RVL‐100‐M, Echo, San Diego, CA, USA).

### 2.17 Single‐cell RNA sequencing (scRNA‐seq) data processing

The raw scRNA‐seq data were obtained from the GSA database under accession code HRA000728, and the data processing and analysis method was described in detail in the previously published article.^46^ Briefly, transcripts were mapped to the corresponding reference genome (GRCh38‐3.0.0 for human) using the 10× Genomics CellRanger pipeline (version 3.1.0).^47^ 10× Genomics CellRanger count generated read count matrices for each sample. Subsequently, the count data were imported into the Seurat R package (version 3.2.0)^47^ for quality control, following the steps below: excluding cells with fewer than 500 detected genes or a mitochondrial gene ratio exceeding 10%, and removing genes expressed in fewer than 5 cells. Normalization was achieved using the LogNormalize method. We employed a one‐sided Wilcoxon rank‐sum test to evaluate the specificity of *ALDH3A1* and *IL‐8* genes within keratocytes.

### 2.18 Quantification and statistical analysis

Statistical analysis was conducted utilizing GraphPad Prism 10 software. All experiments were repeated at least three times, meaning that at least three independent experiments were conducted using cells isolated from different donors. Figure legends provide detailed information on the number of replicates for the representative data (*n* = 3 or *n* = 5), and the specific statistical tests applied. Statistical significance was set as *p < .05, **p < .01, ***p < .001, ****p < .0001.

## 3 RESULTS

### 3.1 IL‐1β promotes keratocyte proliferation

In the pathological microenvironment after corneal injury, the pro‐inflammatory factor IL‐1β is released and influences the cornea's physiological characteristics.^9^, ^48^ To study the proliferation and migration of keratocytes following corneal injury, we subjected keratocytes in vitro to 1 ng/mL IL‐1β^9^, ^48^ for varying durations of 0, 12, and 24 h.

**FIGURE 1:**
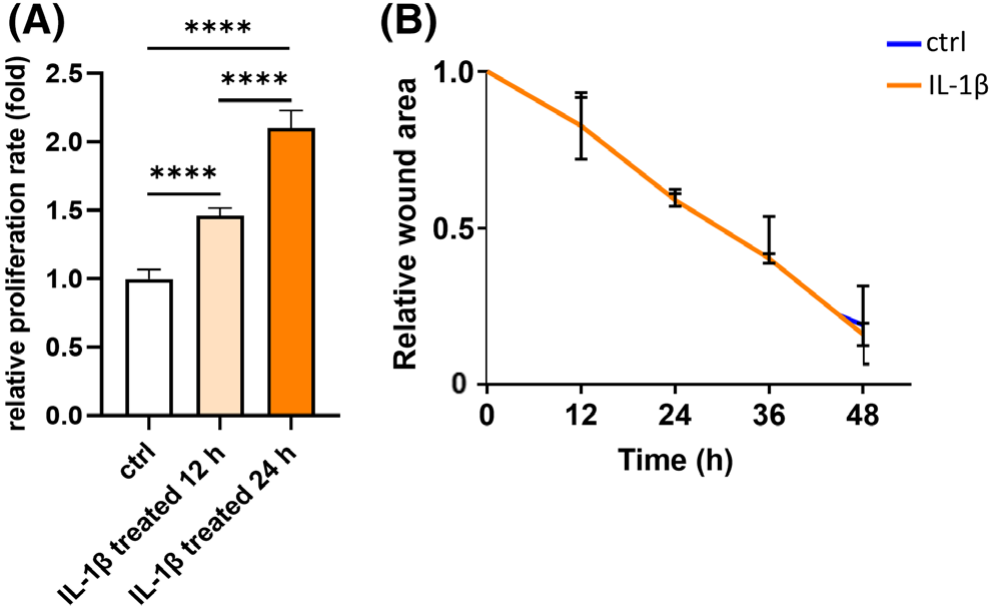
IL‐1β promotes keratocytes proliferation. (A) Keratocytes were exposed to 1 ng/mL IL‐1β for 0 (ctrl), 12, or 24 h, respectively. Proliferation rate was assessed using BrdU assay. *n* = 3. Statistical analyses were conducted by One‐way ANOVA with Tukey's multiple comparisons test. (B) IL‐1𝛽 was administrated when keratocytes were scratched using a 1 mL pipette tip, followed by a 48‐hour incubation. 0, 12, 24, 36 and 48 h post scratching, photographs of the wounded area were captured. The means of the remaining wound area were calculated. Keratocytes without IL‐1β treatment served as the control (ctrl) group. *n* = 3. For each timepoint, statistical analyses were conducted by unpaired *t*‐test. Data is expressed as the mean ± SD. ****p < .0001.

Keratocyte proliferation rate was assessed using BrdU assay. Notably, the proliferation of keratocytes was significantly promoted by IL‐1β in a time‐dependent manner (Figure 1A).

Furthermore, we conducted a scratch wound healing assay on monolayer‐cultured keratocytes to measure migration. IL‐1β was administrated when keratocytes were scratched, followed by a 48‐hour incubation period. At 0, 12, 24, 36 and 48 h post scratching, photographs of the wounded area were captured to monitor the progress of cell migration. Statistical analysis of the remaining wound area indicated that IL‐1β exhibited no significant impact on migration as compared to the control group (Figure 1B).

### 3.2 Strain upregulates ALDH3A1 and inhibits the NF‐κB signaling pathway

Previous results from our group demonstrated that in keratocytes, the activation of the NF‐κB signaling pathway by its inducer IL‐1β leads to a decrease in the mRNA expression of *ALDH3A1*. Meanwhile, the presence of the NF‐κB signaling pathway inhibitor TPCA‐1 reversed the impact of IL‐1β on NF‐κB, subsequently restoring the expression level of ALDH3A1.^9^ IL‐8 is a key downstream product of NF‐κB signaling pathway.^49^, ^50^ The expression of IL‐8 is positively correlated with the activation of NF‐κB signaling pathway by IL‐1β.^9^

To evaluate the impact of mechanical strain on the expression of ALDH3A1 and the NF‐κB signaling pathway in keratocytes, our study commenced with subjecting keratocytes to 3% strain in the Flexcell® Tension System (described in detail in the Section 2 and a previously published paper^17^). We chose 3% strain since our previous study has shown that 3% strain upregulates the expression of the keratocyte biomarkers LUMICAN and KERATOCAN, and contributes to maintaining keratocyte phenotype.^17^, ^28^ After 24‐, 48‐, and 72‐h strain, we applied RT‐qPCR to quantify the expression levels of *ALDH3A1* along with *IL‐8*.

**FIGURE 2:**
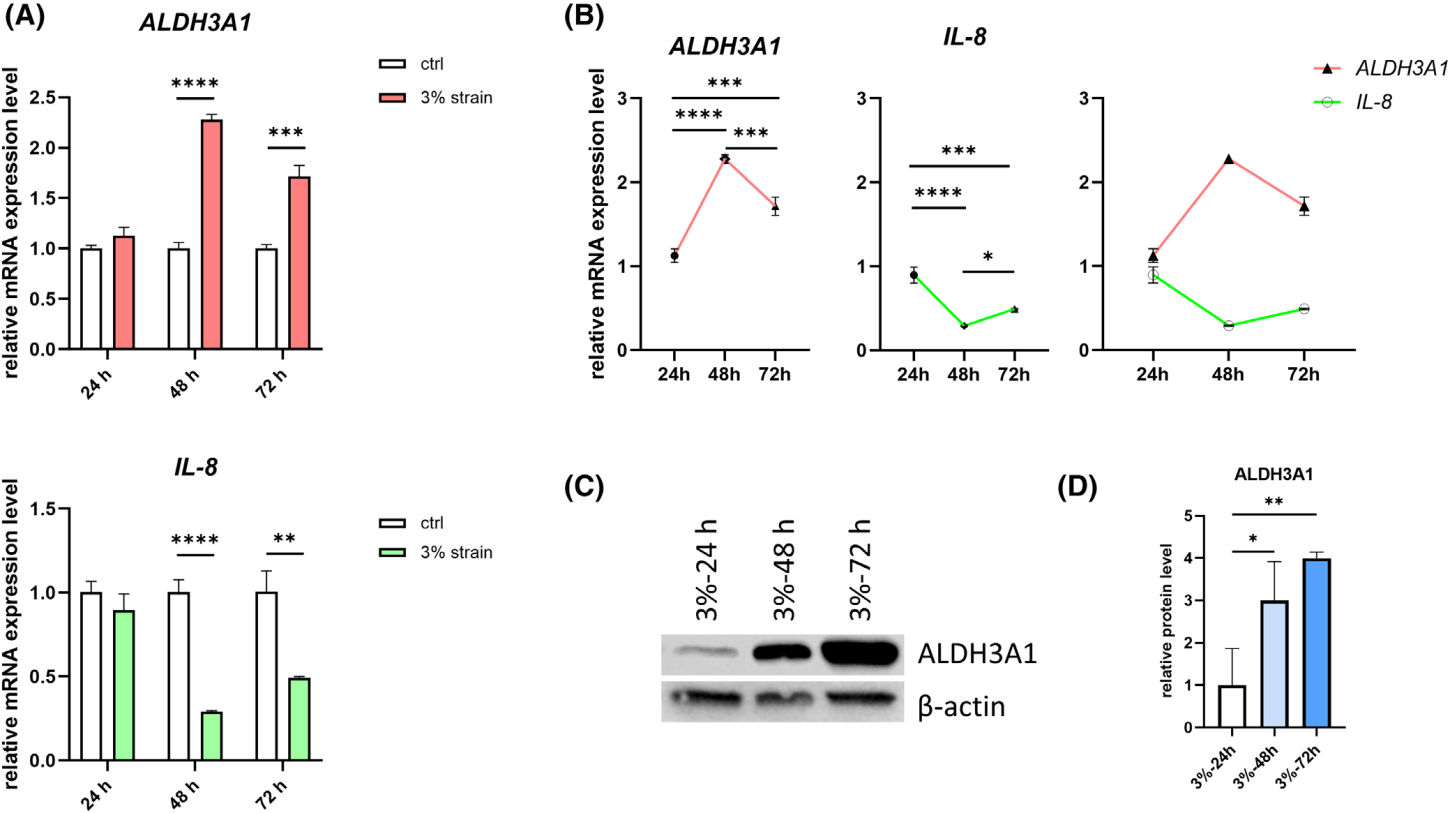
Strain upregulates ALDH3A1 and downregulates IL‐8 expression. Keratocytes were subjected to 3% strain for 24, 48, or 72 h, respectively. Unstrained keratocytes served as control (ctrl) group. (A) The mRNA expression levels of *ALDH3A1* and *IL‐8* were assessed by RT‐qPCR. *n* = 3. For each timepoint, statistical analyses were conducted by unpaired *t*‐test. (B) Time‐dependent mRNA expression pattern of *ALDH3A1* and *IL‐8* in 3% strained keratocytes. *n* = 3. Statistical analyses were conducted by One‐way ANOVA with Tukey's multiple comparisons test. Data were presented as mean ± SD and as target gene expression/*GAPDH*, normalized to the ctrl group. (C) Time‐dependent protein expression levels of ALDH3A1 in 3% strained keratocytes. β‐actin served as a loading control. Representative data from one of three independent western blot experiments conducted using samples from three different donors. (D) The densitometry analysis of the western blot bands of ALDH3A1. *n* = 3. The densitometry of β‐actin bands served as the normalization reference. Statistical analyses were conducted by One‐way ANOVA with Tukey's multiple comparisons test. Data were presented as mean ± SD. *p < .05, **p < .01, ***p < .001, ****p < .0001.

Following 3% strain, the mRNA level of *ALDH3A1* increased in keratocytes. In contrast, the expression of *IL‐8* decreased (Figure 2A). Thus, an intriguing negative correlation emerged in the expression patterns of *ALDH3A1* and *IL‐8*, as illustrated in Figure 2B.

To gain a more comprehensive understanding, we performed western blot to assess the protein levels of ALDH3A1. The outcomes were consistent with those from the RT‐qPCR analysis. Notably, the primary distinction arose in the timing: The peak mRNA level of *ALDH3A1* occurred at the 48‐h time point, whereas the highest protein level manifested at the 72‐h time point (Figure 2C,D). This distinction may arise because, as cells proliferate over time, the newly generated cells grow directly on the already strained membrane, suggesting they may not experience the same level of strain. This lack of strain on the newly generated cells may have contributed to the observed decrease in ALDH3A1 expression at 72 h compared to 48 h at mRNA level. Since the translation of mRNA into protein takes time, this could also explain why ALDH3A1 protein levels peaked at 72 h. However, the expression level of ALDH3A1 remained significantly higher compared to the control group.

**FIGURE 3:**
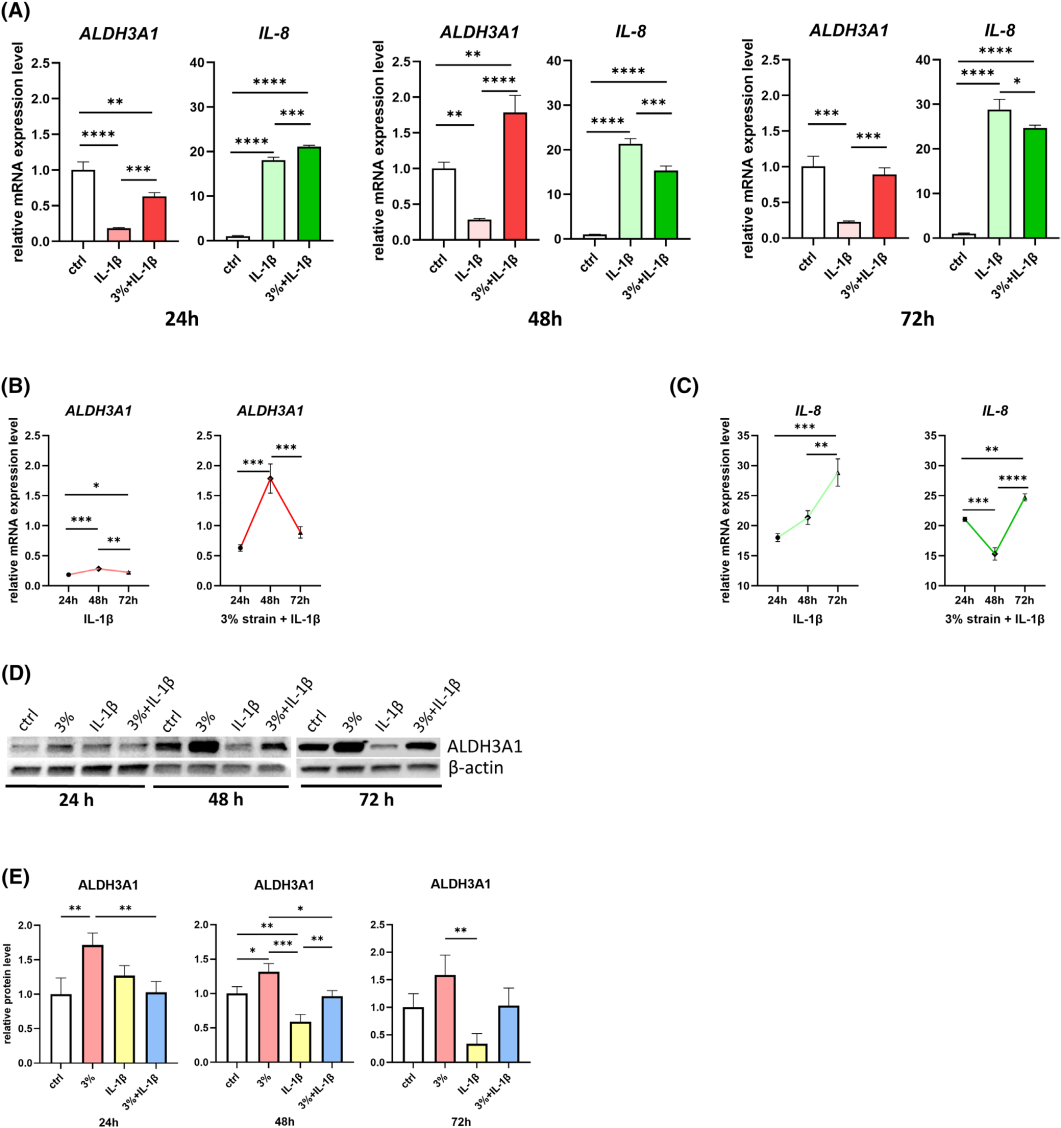
Strain reverses the IL‐1β effects on ALDH3A1 and IL‐8 expression. We administered IL‐1β treatment to keratocytes, which were concurrently subjected to 3% strain over periods of 24, 48, and 72 h. (A) The mRNA expression levels of *ALDH3A1* and *IL‐8* were assessed by RT‐qPCR. *n* = 3. Statistical analyses were conducted by One‐way ANOVA with Tukey's multiple comparisons test. (B, C) Time‐dependent mRNA expression pattern of *ALDH3A1* and *IL‐8* in keratocytes under different treatment conditions. Data were presented as mean ± SD and as target gene expression/*GAPDH*, normalized to the ctrl group. *n* = 3. Statistical analyses were conducted by One‐way ANOVA with Tukey's multiple comparisons test. (D) Protein expression levels of ALDH3A1 in keratocytes under different treatment conditions. β‐actin served as a loading control. Representative data from one of three independent western blot experiments conducted using samples from three different donors. (E) The densitometry analysis of the western blot bands of ALDH3A1. *n* = 3. The densitometry of β‐actin bands served as the normalization reference. Statistical analyses were conducted by One‐way ANOVA with Tukey's multiple comparisons test within each timepoint. Data were presented as mean ± SD. *p < .05, **p < .01, ***p < .001, ****p < .0001.

To verify whether strain could counteract the effects induced by IL‐1β on the NF‐κB signaling pathway and on the expression of ALDH3A1, we administered IL‐1β treatment to keratocytes, which were concurrently subjected to 3% strain over periods of 24, 48, and 72 h. Through RT‐qPCR analysis, we assessed the expression levels of *ALDH3A1* and *IL‐8*. Notably, the decrease in *ALDH3A1* expression induced by IL‐1β was effectively reversed when exposed to 3% strain, consistently across all three time points. Meanwhile, regarding *IL‐8*, the most significant downregulating effect of strain on its expression was observed at the 48‐h time point (Figure 3A).

Examining the effect of IL‐1β on the expression pattern of ALDH3A1 over time, we observed a similarity between conditions with and without strain. However, strain elevated the overall expression level of *ALDH3A1* (Figure 3B). Conversely, the expression pattern of *IL‐8* revealed notable differences between strain and non‐strain conditions (Figure 3C). At the 48‐h time point, a significant decrease of *IL‐8* expression due to strain was observed.

Supporting the above findings, western blot analysis demonstrated that 3% strain exerted an upregulating effect on ALDH3A1 protein expression. Furthermore, the decrease in ALDH3A1 expression induced by IL‐1β was effectively reversed by 3% strain, especially at the 48‐h time points (Figure 3D,E).

These findings imply that 3% strain has the capacity to restore ALDH3A1 expression, effectively counteracting the impact of IL‐1β.

### 3.3 Strain prevents NF‐κB nuclear translocation

Nuclear translocation of NF‐κB is a pivotal step in the activation of the NF‐κB signaling pathway. To verify whether strain could impede NF‐κB nuclear translocation, we subjected keratocytes to concurrent treatment with IL‐1β and 3% strain for 24 h.

**FIGURE 4:**
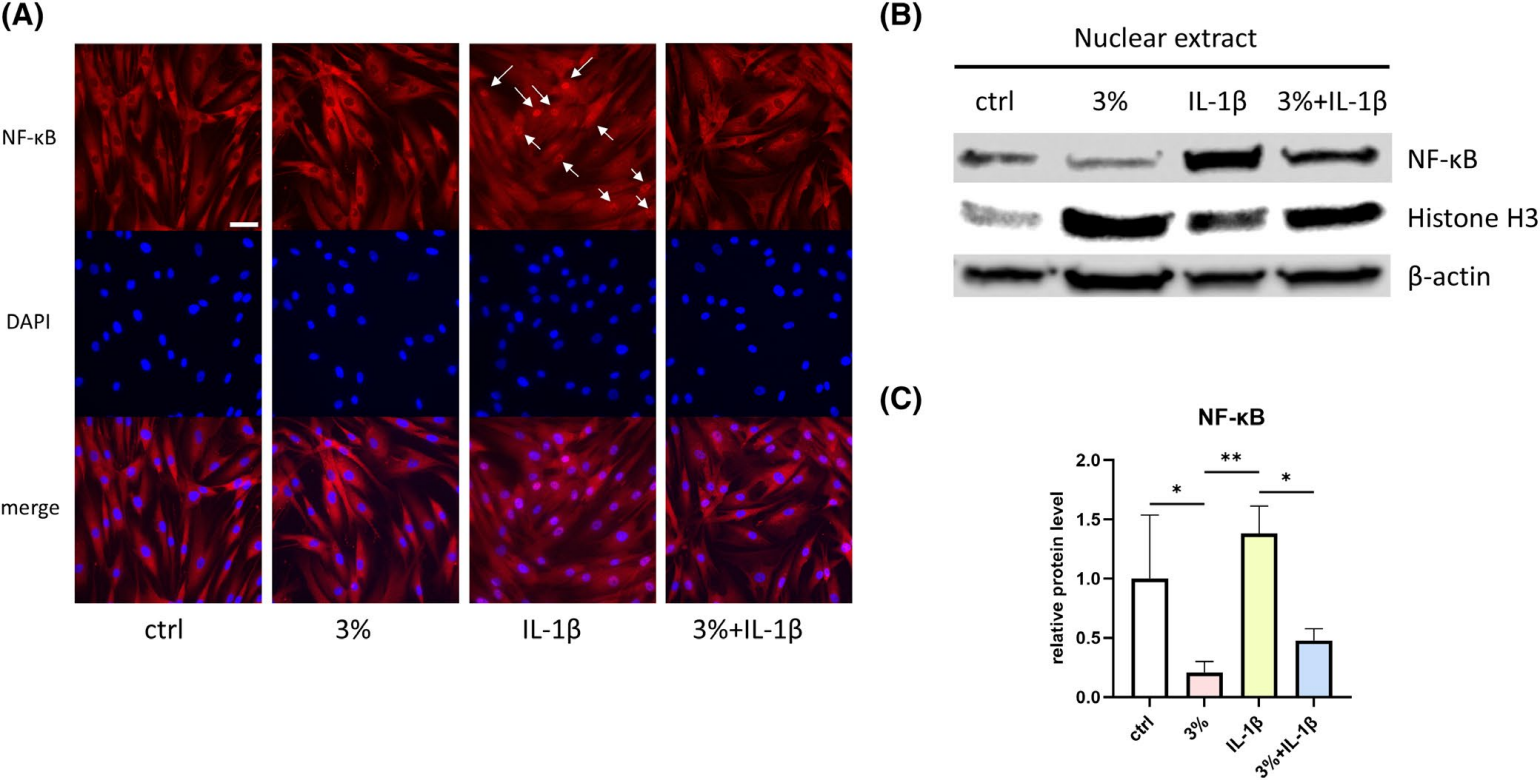
Strain prevents NF‐κB nuclear translocation. Keratocytes were subjected to concurrent treatment with IL‐1β and 3% strain for 24 h. (A) Immunofluorescent staining images of keratocytes. NF‐κB were stained in red, and nuclei were stained in blue. (400‐fold magnification; scale bar, 50 μm) Arrows indicate the nuclear translocation of NF‐κB. Representative data from one of five independent immunofluorescence staining experiments conducted using samples from three different donors. (B) Protein expression levels of NF‐κB in keratocytes nuclear extract. β‐actin served as a loading control for total protein. Histone H3 served as a loading control for nuclear protein. Representative data from one of three independent western blot experiments conducted using samples from three different donors. (C) The densitometry analysis of the western blot bands of NF‐κB. *n* = 3. The densitometry of Histone H3 bands served as the normalization reference. Statistical analyses were conducted by One‐way ANOVA with Tukey's multiple comparisons test within each timepoint. Data were presented as mean ± SD. *p < .05, **p < .01.

As illustrated in Figure 4A, strain effectively prevented NF‐κB nuclear translocation, even in the presence of IL‐1β. Under normal conditions, NF‐κB primarily accumulated within the cytoplasm (Figure 4A, left panel). However, in the presence of IL‐1β, NF‐κB translocated into the nuclei (Figure 4A, third panel from the left, arrows indicate the nuclear translocation of NF‐κB). Notably, when IL‐1β‐treated keratocytes were subjected to 3% strain, the NF‐κB nuclear translocation was reduced (Figure 4A, fourth panel from the left).

Western blot results corroborated the immunofluorescence findings. Under 3% strain, the quantity of NF‐κB in the nuclear extract decreased when comparing 3% strain group to the control group and the 3% strain + IL‐1β group to the IL‐1β only group, respectively, regardless of the presence of IL‐1β (Figure 4B,C).

### 3.4 ALDH3A1 inhibits NF‐κB nuclear translocation

**FIGURE 5:**
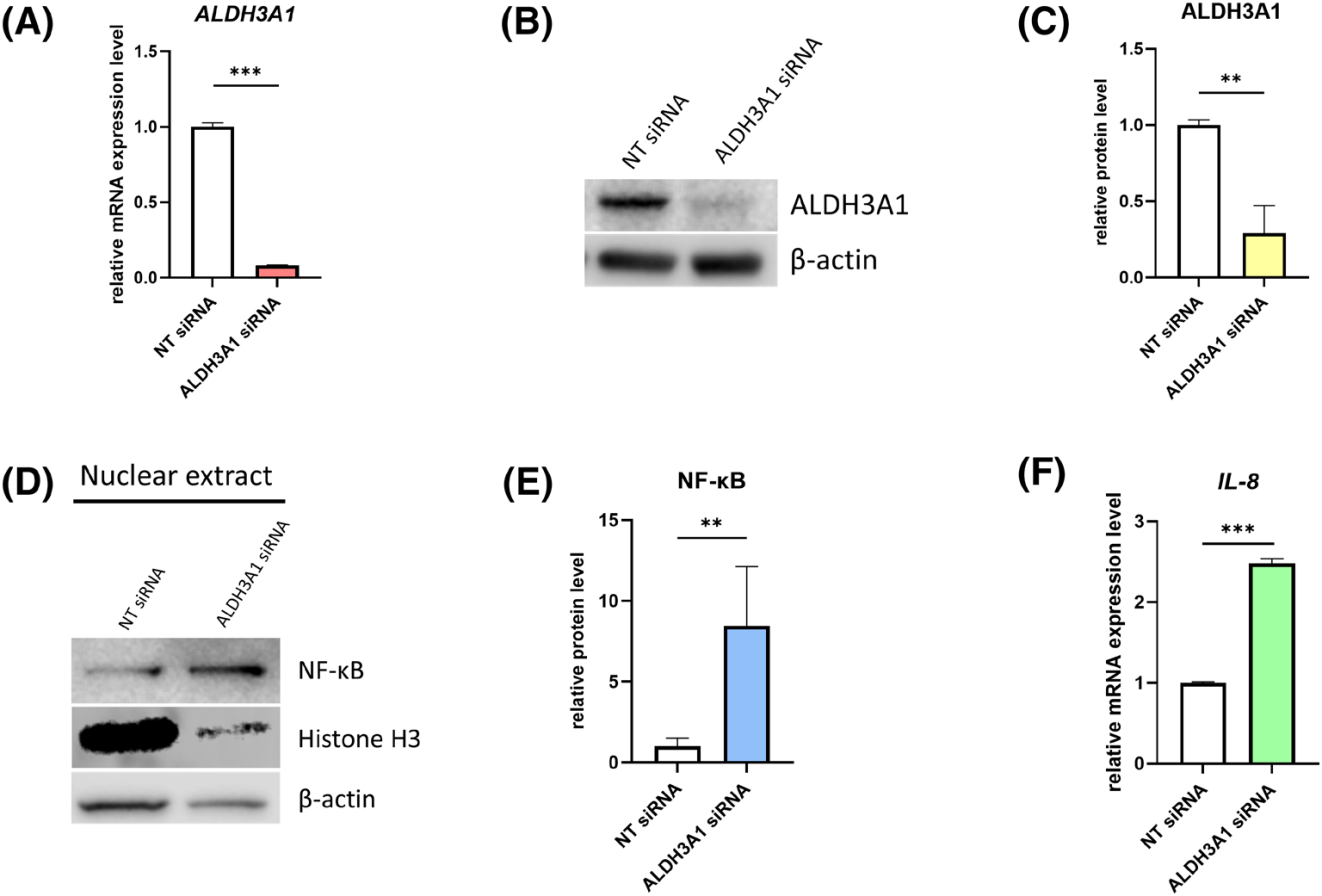
ALDH3A1 inhibits NF‐κB nuclear translocation and further downregulates IL‐8. (A) The mRNA expression levels of *ALDH3A1* were assessed by RT‐qPCR. *n* = 3. Statistical analyses were conducted by unpaired *t*‐test. Data were presented as mean ± SD and as target gene expression/*GAPDH*, normalized to the NT siRNA group. (B) Protein expression level of ALDH3A1 in keratocytes 48 h post transfected with NT siRNA or siRNA targeting ALDH3A1 (ALDH3A1 siRNA). β‐actin served as a loading control. Representative data from one of three independent western blot experiments conducted using samples from three different donors. (C) The densitometry analysis of the western blot bands of ALDH3A1. *n* = 3. The densitometry of β‐actin bands served as normalization. Statistical analyses were conducted by unpaired *t*‐test. Data were presented as mean ± SD. (D) Keratocytes were treated with NT siRNA or ALDH3A1 siRNA for 48 h, following which nuclear extract protein samples were collected, and subjected to western blot analysis. Protein expression level of NF‐κB within the nuclear extract was assessed by western blot β‐actin served as a loading control for total protein. Histone H3 served as a loading control for nuclear protein. Representative data from one of four independent western blot experiments conducted using samples from three different donors. (E) The densitometry analysis of the western blot bands of NF‐κB. *n* = 3. The densitometry of Histone H3 bands served as the normalization reference. Statistical analyses were conducted by unpaired *t*‐test. Data were presented as mean ± SD. (F) The mRNA expression levels of *IL‐8* were assessed by RT‐qPCR. *n* = 3. Statistical analyses were conducted by unpaired *t*‐test. Data were presented as mean ± SD and as target gene expression/*GAPDH*, normalized to the NT siRNA group. **p < .01, ***p < .001.

To establish whether ALDH3A1 could inhibit the activation of the NF‐κB signaling pathway, siRNA targeting ALDH3A1 was utilized to downregulate the expression of ALDH3A1 in keratocytes (Figure 5A–C).

When ALDH3A1 was knocked down, a noticeable increase of NF‐κB within the nuclear extract was observed (Figure 5D,E). Additionally, the downstream product of the NF‐κB signaling pathway, *IL‐8*, increased as *ALDH3A1* was knocked down (Figure 5F).

### 3.5 Knockdown of ALDH3A1 promotes keratocytes migration and proliferation

To study the correlation between ALDH3A1 and keratocytes migration and proliferation, we conducted a scratch wound assay on monolayer‐cultured keratocytes. In this assay, the expression of ALDH3A1 was suppressed using siRNA. Images of the scratched area were captured at 0, 24, and 48 h post‐scratch.

**FIGURE 6:**
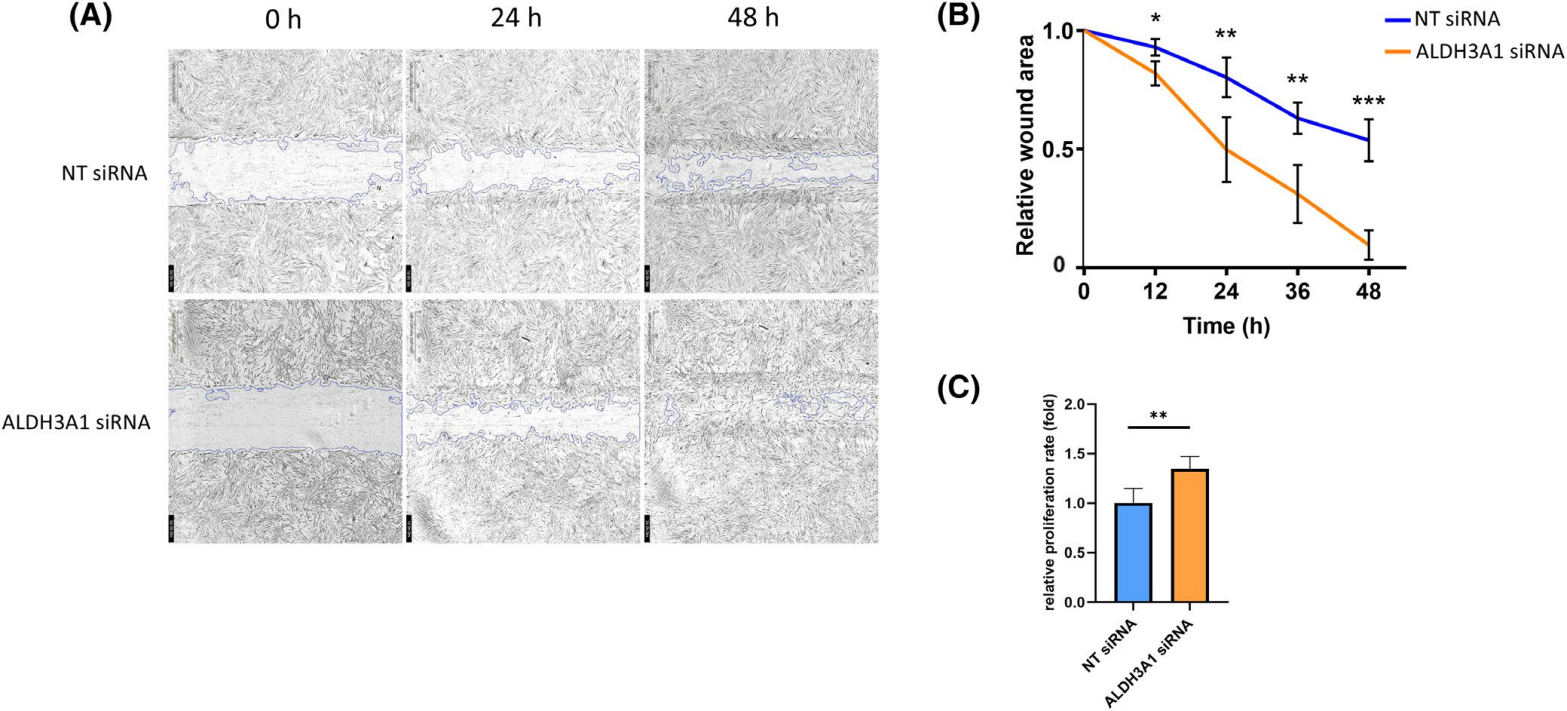
Downregulation of ALDH3A1 promotes keratocyte migration and proliferation. (A) Impact of ALDH3A1 on keratocytes migration. Keratocytes were cultured as a monolayer and treated separately with non‐targeting siRNA (NT siRNA) or siRNA targeting ALDH3A1 (ALDH3A1 siRNA) for 48 h, then scratch‐wounded using a 1 mL pipette tip. Images of the scratched area were captured at 0‐, 24‐, and 48‐h post‐scratch (4‐fold magnification). Representative data from one of three independent experiments conducted using samples from three different donors. (B) The means of the remaining wound area were calculated. *n* = 3. For each timepoint, statistical analyses were conducted by unpaired *t*‐test. (C) Keratocytes were treated separately with non‐targeting siRNA (NT siRNA) or siRNA targeting ALDH3A1 (ALDH3A1 siRNA) for 48 h. The proliferation rate was assessed using BrdU assay. *n* = 3. Statistical analyses were conducted by unpaired *t*‐test. Data is expressed as the mean ± SD. *p < .05, **p < .01, ***p < .001.

Interestingly, keratocytes transfected with ALDH3A1 siRNA displayed a faster closure of the scratched area compared to cells transfected with non‐targeting (NT) siRNA (Figure 6A). Statistical analysis of the remaining scratched area is presented in Figure 6B, showing a significant enhancement in keratocytes migration in the ALDH3A1 siRNA group.

Subsequently, the proliferation rate of keratocytes transfected with ALDH3A1 siRNA was evaluated. The results clearly indicated that the proliferation rate of keratocytes increased in the ALDH3A1 siRNA group (Figure 6C).

### 3.6 Strain suppresses keratocytes proliferation

**FIGURE 7:**
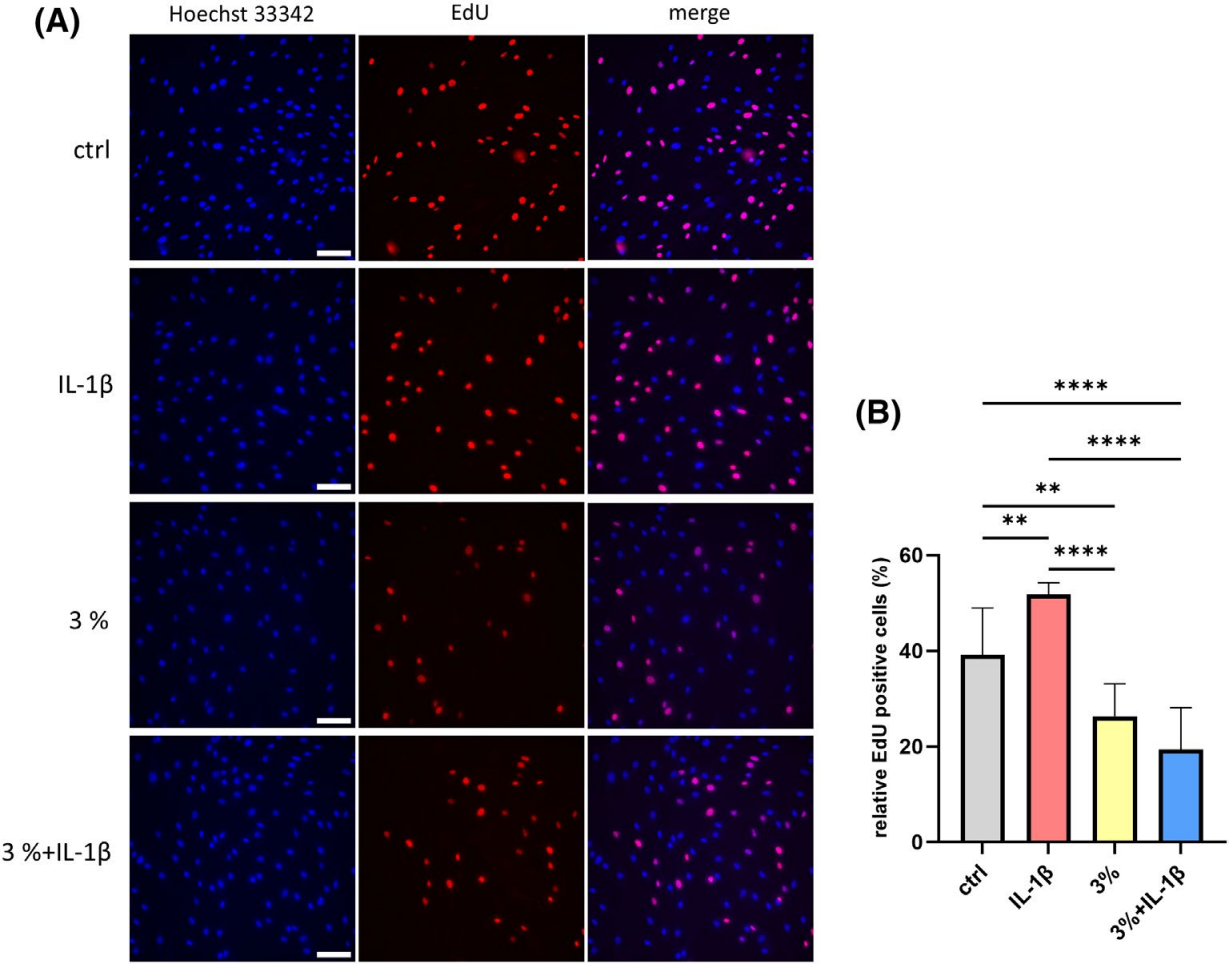
Strain suppresses keratocytes proliferation. (A) EdU staining images of keratocytes. EdU positive cells were stained in red, and nuclei were stained in blue. (200‐fold magnification; scale bar, 100 μm). Representative data from one of three independent EdU staining experiments conducted using samples from three different donors. (B) The statistical analysis of the EdU positive cells. *n* = 9. Statistical analyses were conducted by One‐way ANOVA with Tukey's multiple comparisons test. Data is presented as mean ± SD. **p < .01, ****p < .0001.

To investigate the effect of strain on keratocyte proliferation, cells were subjected to 3% static mechanical strain for 48 h. At the 24‐h point of strain application, 1 ng/mL IL‐1β was added to selected groups. Keratocyte proliferation was then measured using the EdU staining assay. The results showed that 3% strain significantly suppressed keratocyte proliferation compared to the unstrained control group (Figure 7A,B). Moreover, the promotive effect of IL‐1β on keratocyte proliferation was effectively reversed by the 3% strain (Figure 7A,B).

### 3.7 Strain upregulates ALDH3A1 and downregulates IL‐8 in vivo

To validate the influence of strain on ALDH3A1 expression in vivo, we utilized two distinct animal models. In both models, intraocular pressure (IOP) was measured before the injury induction and at 7 days post‐injury.

**FIGURE 8:**
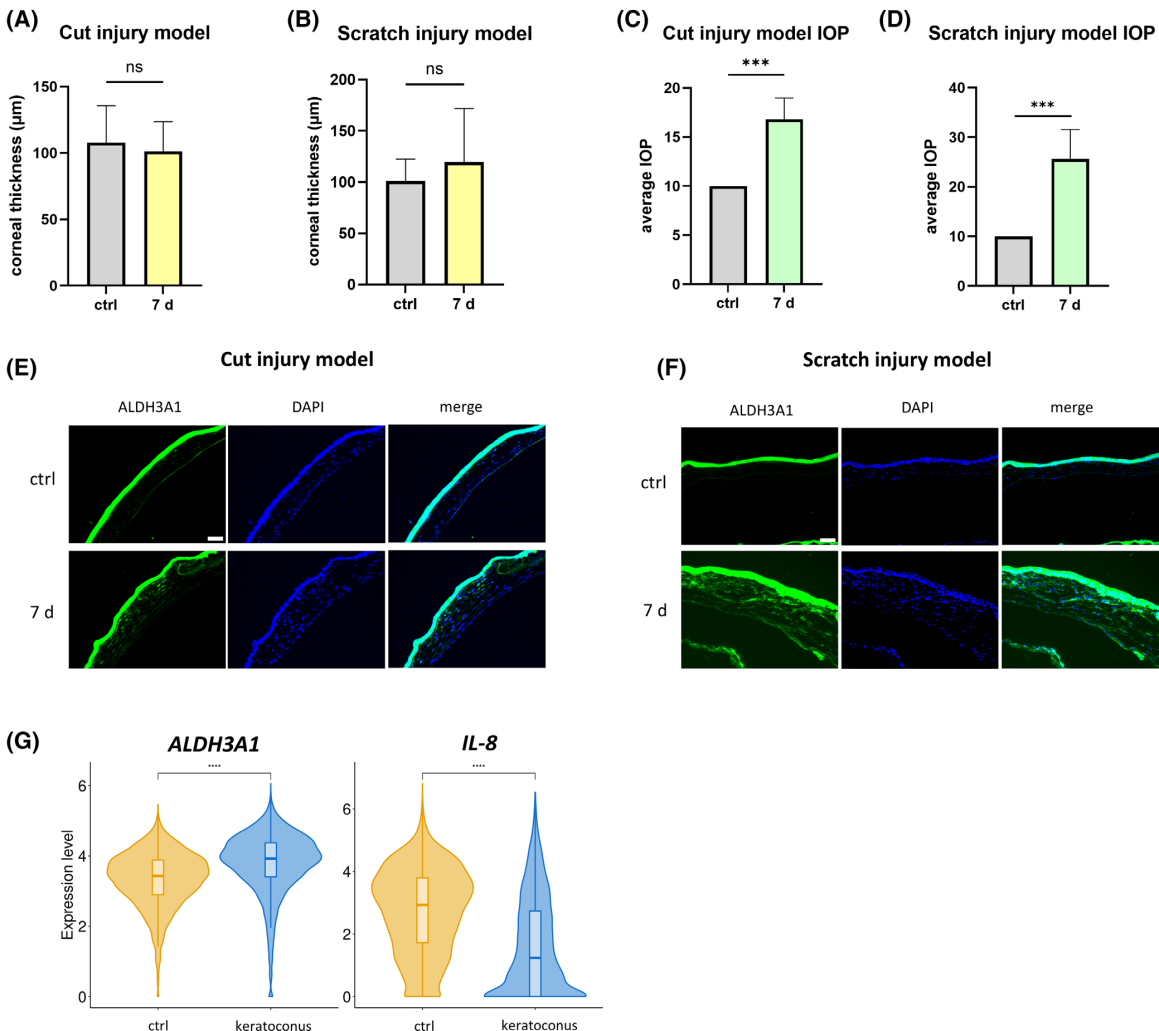
Strain upregulates ALDH3A1 and downregulates IL‐8 in vivo. (A) The corneal thickness of the Cut injury model. *n* = 6. Statistical analyses were conducted by unpaired *t*‐test. Data were presented as mean ± SD. (B) The corneal thickness of the Scratch injury model. *n* = 6. Statistical analyses were conducted by unpaired *t*‐test. Data were presented as mean ± SD. (C) IOP of the Cut injury model. *n* = 5. Statistical analyses were conducted by unpaired *t*‐test. (D) IOP of the Scratch injury model. *n* = 5. Statistical analyses were conducted by unpaired *t*‐test. (E) Immunofluorescent staining images of the cross‐section of the cornea injury site in the Cut injury model. ALDH3A1 were stained in green, and nuclei were stained in blue. (scale bar, 100 μm). Representative data from one of four independent experiments conducted using samples from four different mice. (F) Immunofluorescent staining images of the cross‐section of the cornea injury site in the Scratch injury model. ALDH3A1 were stained in green, and nuclei were stained in blue. (scale bar, 100 μm). Representative data from one of four independent experiments conducted using samples from four different mice. (G) The scRNA‐Seq data of the expression levels of *ALDH3A1* and *IL‐8* in keratocytes from keratoconus patients. Keratocytes were isolated from cornea tissues of 3 keratoconus patients and 4 healthy donors (served as ctrl group). One‐sided Wilcoxon rank‐sum test was performed to assess *ALDH3A1* and *IL‐8* specificity within keratocytes. ns, no significant difference, ***p < .001, ****p < .0001.

Our data showed that there was no significant change in corneal thickness before the injury and 7 days post‐injury in both models (Figure 8A,B). The outcome consistently demonstrated a positive correlation between increased ALDH3A1 expression in the corneal stroma and an increase in IOP, in both models (Figure 7C–F). Previous studies have confirmed that IOP is positively correlated with corneal strain.^51^, ^52^ Elevated IOP levels correspond to an increased force applied to the inner surface of the cornea, subsequently leading to a higher corneal strain. Thus, the above result could be interpreted as ALDH3A1 expression increasing in the corneal stroma following the elevation of corneal strain due to increased IOP in both models.

A consistent reduction in corneal elastic modulus has been observed in the corneal stroma of keratoconus patients.^53^, ^54^ From a biomechanical standpoint, this decrease in corneal elastic modulus leads to a weakening of the resistance to deformation in the cornea.^55^ In the progression of keratoconus, the central cornea protrudes, leading to heightened local strain.^55^, ^56^ To study whether the heightened strain in keratoconus had an impact on the expression of ALDH3A1 and IL‐8, keratocytes were isolated from corneal tissues of 3 keratoconus patients and 4 healthy donors. Subsequently, scRNA‐Seq was conducted using the 10x Genomics platform for each group separately, as described in detail in the Section 2 and a previously published paper.^46^

The scRNA‐Seq revealed a significant increase in the expression levels of *ALDH3A1* in keratocytes from keratoconus patients (Figure 7G, left panel), while the expression level of *IL‐8* demonstrated a decrease (Figure 7G, right panel). These ex vivo results were highly consistent with the findings obtained in our in vitro experiments, thus strongly supporting our hypothesis that strain plays a pivotal role in upregulating ALDH3A1 expression and inhibiting the NF‐κB signaling pathway in human keratocytes.

## 4 DISCUSSION

This study, employing in vitro, ex vivo and in vivo models, demonstrates for the first time that corneal biomechanical properties play a pivotal role in regulating the expression of ALDH3A1 in human keratocytes, thereby modulating its cellular behaviors, including proliferation and migration.

In the event of corneal injury, IL‐1β released from corneal epithelium, along with other cytokines, stimulate keratocytes surrounding the wounded area to initiate proliferation, migrate towards the site of injury, and differentiate into fibroblasts and myofibroblasts.^57^, ^58^ Zieske et al. demonstrated that about 12–24 h after the initial injury, residual keratocytes initiate proliferation.^59^ Other studies have examined the impact of IL‐1β on keratocytes, primarily concentrating on apoptosis and differentiation.^60^, ^61^ Our research revealed that IL‐1β notably promotes keratocyte proliferation, although it does not significantly affect migration. This finding suggests that proliferation following corneal injury is, to some extent, regulated by IL‐1β. Human cornea possesses intricate and distinctive biomechanical characteristics, such as strain, viscoelasticity, non‐linear elasticity, and heterogeneity.^4^ These biomechanical properties are essential for maintaining the normal physiological function of the cornea. Consequently, corneal injury not only triggers the secretion of IL‐1β and other cytokines but also induces notable alterations in its biomechanical properties. Understanding corneal biomechanics is crucial for comprehending the physiological processes of the cornea and for the prevention and treatment of ocular diseases like keratoconus.

In recent years, studies have focused on the impact of ALDH3A1 on cell behavior. Knocking‐down ALDH3A1 expression in alveolar epithelial cells (A549 cells) significantly diminishes their proliferation, migration, and invasion capabilities.^38^ Overexpressing ALDH3A1 in human skin keratinocytes (NCTC 2455) and up‐regulating its expression in epithelial cells from cornea (HCE cell line) to levels similar of those in vivo in humans, prolong proliferation time, reduce colony formation efficiency, and markedly inhibit DNA synthesis, in both cell lines.^34^ Some researchers suggest that ALDH3A1‐mediated inhibition of cell growth in the cornea may aid in repairing oxidative stress‐induced DNA damage and reduce UV‐induced apoptosis.^29^, ^34^ Pappa et al. identified ALDH3A1 as a negative regulator of the corneal epithelial cell cycle, inhibiting proliferation and promoting cell survival.^34^ Our research reveals a close correlation between changes in corneal strain and the regulation of ALDH3A1 expression in human keratocytes. Compared to the unstrained group, a 3% strain significantly increases ALDH3A1 expression at both mRNA and protein levels. This upregulation of ALDH3A1 further inhibits the proliferation and migration of keratocytes. Conversely, when ALDH3A1 expression is knocked down, keratocyte proliferation and migration are markedly enhanced. Moreover, our results indicate that the enhanced migration is more likely attributable to the downregulation of ALDH3A1 rather than the increase in proliferation, since exposure of keratocytes to IL‐1β clearly showed that a mere increase in proliferation does not promote keratocyte migration (Figure 1). The upregulation of ALDH3A1 is confirmed in two mice models. Through single‐cell RNA sequencing analysis of keratocytes from keratoconus patients, we also confirmed the increased expression of ALDH3A1 and the decreased expression of IL‐8. In our models to induce corneal injury in mice, IOP levels increase, and immunofluorescence results reveal an increase in ALDH3A1 expression within the corneal stroma alongside the increased IOP. Similarly, keratoconus patients exhibit prolonged elevation in corneal strain, and our results showed a significant upregulation of ALDH3A1 mRNA in keratocytes confirming a similar correlation as in our two mice models. While IOP and strain are distinct concepts, they are closely linked. Increased IOP can lead to mechanical stress on the cornea, causing it to deform or strain. Our findings support and highlight the pivotal role of corneal biomechanical characteristics in both the maintenance and regulation of corneal physiological function.

Based on our results, we propose that the upregulation of ALDH3A1 expression in keratocytes due to corneal strain may have a corresponding impact on the cell‐mediated immune response within the cornea. Various cell‐mediated immune responses play a role in corneal stromal wound healing. As part of this process, immune cells infiltrate the wound site to remove cellular debris and prevent infection. In this process, IL‐1β serves as a crucial mediator in cell‐mediated immunity by activating and modulating various immune cells and creating an inflammatory environment that supports the development of adaptive immune responses.^62^, ^63^ Previous research^64^ has shown that ALDH3A1 overexpression reprograms the cells' surrounding microenvironment, leading to a reduced proliferation of peripheral blood mononuclear cells, which suggests a suppressive effect of ALDH3A1 on immune cell function. Our findings show that corneal strain upregulates ALDH3A1 expression in keratocytes, and this upregulation may reprogram the surrounding microenvironment of keratocytes, thereby inhibiting immune cell proliferation and infiltration into the corneal stroma, ultimately modulating the immune microenvironment of the corneal stroma. At the same time, the reverse effect of 3% strain on IL‐1β suggests that corneal strain plays a regulatory role in IL‐1β‐induced cell‐mediated immunity.

It is widely recognized that the nuclear translocation of NF‐κB plays an essential role in mediating a broad range of external stimuli to the transcriptional activation of specific target genes and in initiating production of inflammatory cytokines.^65^, ^66^ Studies have demonstrated that inflammation significantly delays the repair process of corneal injury.^67^, ^68^ Our study observes that a 3% strain, when compared to the unstrained group, inhibits the activation of the NF‐κB signaling pathway by impeding NF‐κB nuclear translocation, consequently affecting the generation of IL‐8, a critical downstream product of NF‐κB signaling pathway. This inhibition is closely associated with the upregulation of ALDH3A1 expression. Increased ALDH3A1 expression induced by 3% strain inhibits NF‐κB nuclear translocation and significantly reduces IL‐8 production. This result of strain is effective even in the presence of IL‐1β, a strong activator of the NF‐κB signaling pathway. Conversely, siRNA‐mediated knockdown of ALDH3A1 leads to enhanced NF‐κB nuclear translocation, coupled with a notable increase in IL‐8 expression. IL‐8 is recognized as a key mediator of inflammation in the cornea.^69^, ^70^ Therefore, our findings suggest that corneal strain may influence the corneal wound healing process by modulating inflammation. Following corneal injury, alterations in ALDH3A1 expression due to changes in corneal strain impact the activation of the NF‐κB signaling pathway and the secretion of its downstream cytokines such as IL‐8, thereby modulating inflammatory processes in the cornea. To support this suggestion, our analysis of keratocytes from keratoconus patients reveals that prolonged elevated strain increases ALDH3A1 expression while significantly inhibiting IL‐8 expression, consistent with our in vitro findings. Notably, combined with previous studies from our group, IL‐1β has been shown to downregulate ALDH3A1 expression by activating the NF‐κB signaling pathway, indicating a mutual regulatory correlation between ALDH3A1 expression and NF‐κB pathway activation.

A significant factor contributing to scar formation after corneal injury is the abnormal proliferation and migration of keratocytes to the wounded area, where they transform into myofibroblasts and continually generate ECM. This overproduction of ECM disrupts the normal structure of the corneal stroma, ultimately leading to corneal scar formation. NF‐κB nuclear translocation is a crucial step in the activation of NF‐κB signaling pathway.^65^ It is widely recognized that the activation of the NF‐κB signaling pathway plays a pivotal role in promoting both cell proliferation and migration. It regulates the expression of genes involved in cell survival, growth, and movement, as well as factors related to invasion, thereby facilitating both cell proliferation and migration.^71^, ^72^, ^73^ Moreover, the inflammatory response triggered by the activation of NF‐κB signaling pathway is also a primary factor affecting the normal progression of corneal wound healing. Our study shows that ALDH3A1 knockdown enhances NF‐κB nuclear translocation, thereby activating NF‐κB signaling pathway and promoting keratocyte proliferation and migration. Strain inhibits the proliferation and migration of keratocytes by upregulating the expression of ALDH3A1, which is closely coordinated with the NF‐κB nuclear translocation. This regulation helps to prevent abnormal keratocyte proliferation and migration, thereby reducing the risk of excessive ECM production, which could otherwise lead to corneal scar formation. Our findings enhance the understanding of the regulatory mechanisms governing keratocyte phenotype and cell behavior, and consequently offer a new perspective for the development of future treatment or improvement of corneal scarring or other diseases resulting from abnormal corneal wound healing processes. We are particularly interested in elucidating the mechanism by which strain modulates the expression of ALDH3A1, and this aspect will be one of the focuses in our future studies.

## AUTHOR CONTRIBUTIONS

*Conceptualization*: Qian Zhang, Xin Zhou, Ludvig J. Backman, and Patrik Danielson. *Methodology and investigation*: Qian Zhang, Xin Zhou, Xiaolei Wang, Shengqian Dou, Leilei Zhao, and Roine El‐Habta. *Writing—original draft*: Qian Zhang. *Writing—review & editing*: Qian Zhang, Xin Zhou, Ludvig J. Backman, and Patrik Danielson. *Supervision and funding*: Ludvig J. Backman, Patrik Danielson, Qian Zhang, and Wei Zhang.

## FUNDING INFORMATION

This work was supported by the Swedish Research Council (Grant 2017‐01138), the foundation Kronprinsessan Margaretas Arbetsnamnd for synskadade (KMA, Grant 2013/10), National Key Research and Development Program of China (2023YFE0206700) and via Federal funds through a regional agreement (ALF) between Umea University and Region Vasterbotten (RV‐979985).

## DISCLOSURES

The authors declare no conflicts of interest.

## Supporting information


Figure S1.

Table S1.

## Data Availability

All data reported in this paper will be shared upon reasonable request by the lead contact, Ludvig Backman (ludvig.backman@umu.se). Any additional information required to reanalyze the data reported in this work paper is available from the lead contact upon request. The data that support the findings of this study are available in the Sections 2 and 3.

## References

[1] Hovakimyan M , Falke K , Stahnke T , et al. Morphological analysis of quiescent and activated keratocytes: a review of ex vivo and in vivo findings. Curr Eye Res. 2014;39:1129‐1144. 24749788 10.3109/02713683.2014.902073

[2] Yam GHF , Riau AK , Funderburgh ML , Mehta JS , Jhanji V . Keratocyte biology. Exp Eye Res. 2020;196:108062. 32442558 10.1016/j.exer.2020.108062

[3] Mlyniuk P , Maczynska‐Walkowiak E , Rzeszewska‐Zamiara J , Grulkowski I , Kaluzny BJ . Probing biomechanical properties of the cornea with air‐puff‐based techniques ‐ an overview. Adv Opt Technol. 2021;10:375‐391.

[4] Blackburn BJ , Jenkins MW , Rollins AM , Dupps WJ . A review of structural and biomechanical changes in the cornea in aging, disease, and photochemical crosslinking. Front Bioeng Biotechnol. 2019;7:66. 31019909 10.3389/fbioe.2019.00066 PMC6459081

[5] Meek KM , Knupp C . Corneal structure and transparency. Prog Retin Eye Res. 2015;49:1‐16. 26145225 10.1016/j.preteyeres.2015.07.001 PMC4655862

[6] Vrehen AF , Rutten M , Dankers PYW . Development of a fully synthetic corneal stromal construct via supramolecular hydrogel engineering. Adv Healthc Mater. 2023;12:e2301392. 37747759 10.1002/adhm.202301392 PMC11468521

[7] Petroll WM , Miron‐Mendoza M , Sunkara Y , Ikebe HR , Sripathi NR , Hassaniardekani H . The impact of UV cross‐linking on corneal stromal cell migration, differentiation and patterning. Exp Eye Res. 2023;233:109523. 37271309 10.1016/j.exer.2023.109523 PMC10825899

[8] Mohan RR , Kempuraj D , D'Souza S , Ghosh A . Corneal stromal repair and regeneration. Prog Retin Eye Res. 2022;91:101090. 35649962 10.1016/j.preteyeres.2022.101090 PMC11926992

[9] Zhou X , Li JH , Backman LJ , Danielson P . Keratocyte differentiation is regulated by NF‐kappa B and TGF beta signaling crosstalk. Int J Mol Sci. 2022;23:11073. 36232373 10.3390/ijms231911073 PMC9570283

[10] Formisano N , van der Putten C , Grant R , et al. Mechanical properties of bioengineered corneal stroma. Adv Healthc Mater. 2021;10:e2100972. 34369098 10.1002/adhm.202100972 PMC11468718

[11] Kamil S , Mohan RR . Corneal stromal wound healing: major regulators and therapeutic targets. Ocul Surf. 2021;19:290‐306. 33127599 10.1016/j.jtos.2020.10.006 PMC9183221

[12] Netto MV , Mohan RR , Ambrosio R Jr , Hutcheon AE , Zieske JD , Wilson SE . Wound healing in the cornea: a review of refractive surgery complications and new prospects for therapy. Cornea. 2005;24:509‐522. 15968154 10.1097/01.ico.0000151544.23360.17

[13] Stramer BM , Zieske JD , Jung JC , Austin JS , Fini ME . Molecular mechanisms controlling the fibrotic repair phenotype in cornea: implications for surgical outcomes. Invest Ophthalmol Vis Sci. 2003;44:4237‐4246. 14507867 10.1167/iovs.02-1188

[14] Shin TJ , Vito RP , Johnson LW , McCarey BE . The distribution of strain in the human cornea. J Biomech. 1997;30:497‐503. 9109561 10.1016/s0021-9290(97)84433-8

[15] Kling S , Khodadadi H , Goksel O . Optical coherence elastography‐based corneal strain imaging during low‐amplitude intraocular pressure modulation. Front Bioeng Biotechnol. 2019;7:453. 32083064 10.3389/fbioe.2019.00453 PMC7004960

[16] Medina A . Plastic modification of the cornea by pneumatic force corrects myopia: pneumatic keratology. Eye (Lond). 2017;31:1621‐1627. 28622329 10.1038/eye.2017.123 PMC5684457

[17] Zhang W , Chen JL , Backman LJ , Malm AD , Danielson P . Surface topography and mechanical strain promote Keratocyte phenotype and extracellular matrix formation in a biomimetic 3D corneal model. Adv Healthc Mater. 2017;6:1601238. 10.1002/adhm.201601238 28026154

[18] Kling S , Hafezi F . Corneal biomechanics ‐ a review. Ophthalmic Physiol Opt. 2017;37:240‐252. 28125860 10.1111/opo.12345

[19] Hollman KW , Emelianov SY , Neiss JH , et al. Strain imaging of corneal tissue with an ultrasound elasticity microscope. Cornea. 2002;21:68‐73. 11805511 10.1097/00003226-200201000-00015

[20] Petroll WM , Vishwanath M , Ma L . Corneal fibroblasts respond rapidly to changes in local mechanical stress. Invest Ophthalmol Vis Sci. 2004;45:3466‐3474. 15452051 10.1167/iovs.04-0361

[21] Morishige N , Magome K , Ueno A , Matsui TA , Nishida T . Relations among corneal curvature, thickness, and volume in Keratoconus as evaluated by anterior segment‐optical coherence tomography. Invest Ophthalmol Vis Sci. 2019;60:3794‐3802. 31525776 10.1167/iovs.19-27619

[22] Karimi A , Razaghi R , Girkin CA , Downs JC . Ocular biomechanics due to ground blast reinforcement. Comput Meth Prog Bio. 2021;211:106425. 10.1016/j.cmpb.2021.106425 PMC8577623 34598082

[23] Takahashi R , Okamura K , Tsukahara‐Kawamura T , et al. Finite element analysis of changes in tensile strain by airsoft gun impact on eye and deformation rate in eyes of various axial lengths. Clin Ophthalmol. 2020;14:1445‐1450. 32546952 10.2147/OPTH.S249483 PMC7266397

[24] Zhang L , Liang L , Su T , et al. Regulation of the keratocyte phenotype and cell behavior derived from human induced pluripotent stem cells by substrate stiffness. ACS Biomater Sci Eng. 2023;9:856‐868. 36668685 10.1021/acsbiomaterials.2c01003

[25] Chen J , Backman LJ , Zhang W , Ling C , Danielson P . Regulation of keratocyte phenotype and cell behavior by substrate stiffness. ACS Biomater Sci Eng. 2020;6:5162‐5171. 33455266 10.1021/acsbiomaterials.0c00510

[26] Chen J , Mo Q , Sheng R , et al. Stiffness‐dependent dynamic effect of inflammation on keratocyte phenotype and differentiation. Biomed Mater. 2023;18:045001. 10.1088/1748-605X/accda9 37068490

[27] Petroll WM , Lakshman N . Fibroblastic transformation of corneal keratocytes by Rac inhibition is modulated by extracellular matrix structure and stiffness. J Funct Biomater. 2015;6:222‐240. 25874856 10.3390/jfb6020222 PMC4493509

[28] Chen JL , Zhang W , Backman LJ , Kelk P , Danielson P . Mechanical stress potentiates the differentiation of periodontal ligament stem cells into keratocytes. Br J Ophthalmol. 2018;102:562‐569. 29306866 10.1136/bjophthalmol-2017-311150 PMC5890647

[29] Estey T , Piatigorsky J , Lassen N , Vasiliou V . ALDH3A1: a corneal crystallin with diverse functions. Exp Eye Res. 2007;84:3‐12. 16797007 10.1016/j.exer.2006.04.010

[30] Chen Y , Thompson DC , Koppaka V , Jester JV , Vasiliou V . Ocular aldehyde dehydrogenases: protection against ultraviolet damage and maintenance of transparency for vision. Prog Retin Eye Res. 2013;33:28‐39. 23098688 10.1016/j.preteyeres.2012.10.001 PMC3570594

[31] Piatigorsky J . Enigma of the abundant water‐soluble cytoplasmic proteins of the cornea: the “refracton” hypothesis. Cornea. 2001;20:853‐858. 11685065 10.1097/00003226-200111000-00015

[32] Voulgaridou GP , Tsochantaridis I , Tolkas C , et al. Aldehyde dehydrogenase 3A1 confers oxidative stress resistance accompanied by altered DNA damage response in human corneal epithelial cells. Free Radic Biol Med. 2020;150:66‐74. 32006654 10.1016/j.freeradbiomed.2020.01.183

[33] Voulgaridou GP , Theologidis V , Venetikidou M , et al. Investigating the functional roles of aldehyde dehydrogenase 3A1 in human corneal epithelial cells. Int J Mol Sci. 2023;24:5845. 36982917 10.3390/ijms24065845 PMC10056195

[34] Pappa A , Brown D , Koutalos Y , DeGregori J , White C , Vasiliou V . Human aldehyde dehydrogenase 3A1 inhibits proliferation and promotes survival of human corneal epithelial cells. J Biol Chem. 2005;280:27998‐28006. 15905174 10.1074/jbc.M503698200

[35] Oraldi M , Saracino S , Maggiora M , et al. Importance of inverse correlation between ALDH3A1 and PPAR gamma in tumor cells and tissue regeneration. Chem Biol Interact. 2011;191:171‐176. 21251908 10.1016/j.cbi.2011.01.011

[36] Moreb JS , Baker HV , Chang LJ , et al. ALDH isozymes downregulation affects cell growth, cell motility and gene expression in lung cancer cells. Mol Cancer. 2008;7:87. 19025616 10.1186/1476-4598-7-87 PMC2605459

[37] Canuto RA , Muzio G , Salvo RA , et al. The effect of a novel irreversible inhibitor of aldehyde dehydrogenases 1 and 3 on tumour cell growth and death. Chem Biol Interact. 2001;130:209‐218. 11306045 10.1016/s0009-2797(00)00280-5

[38] Fan FF , Yin RX , Wang LY , et al. ALDH3A1 driving tumor metastasis is mediated by p53/BAG1 in lung adenocarcinoma. J Cancer. 2021;12:4780‐4790. 34234849 10.7150/jca.58250 PMC8247369

[39] Pei Y , Reins RY , McDermott AM . Aldehyde dehydrogenase (ALDH) 3A1 expression by the human keratocyte and its repair phenotypes. Exp Eye Res. 2006;83:1063‐1073. 16822507 10.1016/j.exer.2006.05.011

[40] Lynch AP , O'Sullivan F , Ahearne M . The effect of growth factor supplementation on corneal stromal cell phenotype in vitro using a serum‐free media. Exp Eye Res. 2016;151:26‐37. 27456135 10.1016/j.exer.2016.07.015

[41] Qu Y , He Y , Yang Y , et al. ALDH3A1 acts as a prognostic biomarker and inhibits the epithelial mesenchymal transition of oral squamous cell carcinoma through IL‐6/STAT3 signaling pathway. J Cancer. 2020;11:2621‐2631. 32201532 10.7150/jca.40171 PMC7066020

[42] Sloniecka M , Le Roux S , Boman P , Byström B , Zhou QJ , Danielson P . Expression profiles of neuropeptides, neurotransmitters, and their receptors in human keratocytes in vitro and in situ. PLoS One. 2015;10:e0134157. 26214847 10.1371/journal.pone.0134157 PMC4516240

[43] Suarez‐Arnedo A , Torres Figueroa F , Clavijo C , Arbelaez P , Cruz JC , Munoz‐Camargo C . An image J plugin for the high throughput image analysis of in vitro scratch wound healing assays. PLoS One. 2020;15:e0232565. 32722676 10.1371/journal.pone.0232565 PMC7386569

[44] Qu M , Zhang X , Hu X , et al. BRD4 inhibitor JQ1 inhibits and reverses mechanical injury‐induced corneal scarring. Cell Death Discov. 2018;4:5. 10.1038/s41420-018-0066-1 PMC6060126 30062054

[45] Mittal SK , Omoto M , Amouzegar A , et al. Restoration of corneal transparency by mesenchymal stem cells. Stem Cell Rep. 2016;7:583‐590. 10.1016/j.stemcr.2016.09.001 PMC5063582 27693426

[46] Dou SQ , Wang Q , Zhang B , et al. Single‐cell atlas of keratoconus corneas revealed aberrant transcriptional signatures and implicated mechanical stretch as a trigger for keratoconus pathogenesis. Cell Discov. 2022;8:79. 35961981 10.1038/s41421-022-00452-9 PMC9374743

[47] Satija R , Farrell JA , Gennert D , Schier AF , Regev A . Spatial reconstruction of single‐cell gene expression data. Nat Biotechnol. 2015;33:495‐502. 25867923 10.1038/nbt.3192 PMC4430369

[48] Chaurasia SS , Lim RR , Lakshminarayanan R , Mohan RR . Nanomedicine approaches for corneal diseases. J Funct Biomater. 2015;6:277‐298. 25941990 10.3390/jfb6020277 PMC4493512

[49] Sloniecka M , Le Roux S , Zhou QJ , Danielson P . Substance P enhances keratocyte migration and neutrophil recruitment through interleukin‐8. Mol Pharmacol. 2016;89:215‐225. 26646648 10.1124/mol.115.101014

[50] Zhou X , Backman LJ , Danielson P . Activation of NF‐κB signaling via cytosolic mitochondrial RNA sensing in kerotocytes with mitochondrial DNA common deletion. Sci Rep. 2021;11:7360. 33795727 10.1038/s41598-021-86522-6 PMC8016944

[51] Kenia VP , Kenia RV , Pirdankar OH . Age‐related variation in corneal biomechanical parameters in healthy Indians. Indian J Ophthalmol. 2020;68:2921‐2929. 33229671 10.4103/ijo.IJO_2127_19 PMC7856994

[52] Liu GH , Rong H , Pei RX , et al. Age distribution and associated factors of cornea biomechanical parameter stress‐strain index in Chinese healthy population. BMC Ophthalmol. 2020;20:436. 33143686 10.1186/s12886-020-01704-6 PMC7607623

[53] Andreassen TT , Simonsen AH , Oxlund H . Biomechanical properties of keratoconus and normal corneas. Exp Eye Res. 1980;31:435‐441. 7449878 10.1016/s0014-4835(80)80027-3

[54] Scarcelli G , Besner S , Pineda R , Kalout P , Yun SH . In vivo biomechanical mapping of normal and keratoconus corneas. JAMA Ophthalmol. 2015;133:480‐482. 25611213 10.1001/jamaophthalmol.2014.5641 PMC4698984

[55] Brad H , Feldman AJVG , Birdsong O , Reddy V , Saad A , Warren N . ICRS: Corneal Biomechanics Effects. American Academy of Ophthalmology; 2023. https://eyewiki.aao.org/w/index.php?title=ICRS:_Corneal_Biomechanics_Effects&oldid=88498

[56] Roy AS , Shetty R , Kummelil MK . Keratoconus: a biomechanical perspective on loss of corneal stiffness. Indian J Ophthalmol. 2013;61:392‐393. 23925321 10.4103/0301-4738.116057 PMC3775071

[57] Wilson SE . Interleukin‐1 and transforming growth factor beta: commonly opposing, but sometimes supporting, master regulators of the corneal wound healing response to injury. Invest Ophthalmol Vis Sci. 2021;62:8. 10.1167/iovs.62.4.8 PMC8039470 33825855

[58] Wilson SE , Esposito A . Focus on molecules: interleukin‐1: a master regulator of the corneal response to injury. Exp Eye Res. 2009;89:124‐125. 19254714 10.1016/j.exer.2009.02.011 PMC2768564

[59] Zieske JD , Guimaräes SR , Hutcheon AEK . Kinetics of keratocyte proliferation in response to epithelial debridement. Exp Eye Res. 2001;72:33‐39. 11133180 10.1006/exer.2000.0926

[60] Wilson SE , He YG , Weng J , et al. Epithelial injury induces keratocyte apoptosis: hypothesized role for the interleukin‐1 system in the modulation of corneal tissue organization and wound healing. Exp Eye Res. 1996;62:325‐337. 8795451 10.1006/exer.1996.0038

[61] Mohan RR , Liang QW , Kim WJ , Helena MC , Baerveldt F , Wilson SE . Apoptosis in the cornea: further characterization of Fas/Fas ligand system. Exp Eye Res. 1997;65:575‐589. 9464190 10.1006/exer.1997.0371

[62] Yan CX , Gao N , Sun HJ , et al. Targeting imbalance between IL‐1β and IL‐1 receptor antagonist ameliorates delayed epithelium wound healing in diabetic mouse corneas. Am J Pathol. 2016;186:1466‐1480. 27109611 10.1016/j.ajpath.2016.01.019 PMC4901143

[63] Poon MW , Jiang D , Qin P , et al. Inhibition of NUCKS facilitates corneal recovery following alkali burn. Sci Rep‐UK. 2017;7:41224. 10.1038/srep41224 PMC5247723 28106169

[64] Terzuoli E , Bellan C , Aversa S , et al. ALDH3A1 overexpression in melanoma and lung tumors drives cancer stem cell expansion, impairing immune surveillance through enhanced PD‐L1 output. Cancer. 2019;11:1963. 10.3390/cancers11121963 PMC6966589 31817719

[65] Oeckinghaus A , Ghosh S . The NF‐κB family of transcription factors and its regulation. Cold Spring Harb Perspect Biol. 2009;1:a000034. 20066092 10.1101/cshperspect.a000034 PMC2773619

[66] Kawai T , Akira S . Toll‐like receptors and their crosstalk with other innate receptors in infection and immunity. Immunity. 2011;34:637‐650. 21616434 10.1016/j.immuni.2011.05.006

[67] Khalili H , Lee RW , Khaw PT , Brocchini S , Dick AD , Copland DA . An anti‐TNF‐alpha antibody mimetic to treat ocular inflammation. Sci Rep. 2016;6:36905. 27874029 10.1038/srep36905 PMC5118814

[68] Zhao WB , He XZ , Liu RL , Ruan QG . Accelerating corneal wound healing using exosome‐mediated targeting of NF‐κB c‐Rel. Inflamm Regen. 2023;43:6. 36703231 10.1186/s41232-023-00260-y PMC9881367

[69] Strieter RM , Kunkel SL , Elner VM , et al. Interleukin‐8—a corneal factor that induces neovascularization. Am J Pathol. 1992;141:1279‐1284. 1281615 PMC1886757

[70] Ghasemi H , Ghazanfari T , Yaraee R , Faghihzadeh S , Hassan ZM . Roles of IL‐8 in ocular inflammations: a review. Ocul Immunol Inflamm. 2011;19:401‐412. 22106907 10.3109/09273948.2011.618902

[71] Karin M , Cao YX , Greten FR , Li ZW . NF‐κB in cancer: from innocent bystander to major culprit. Nat Rev Cancer. 2002;2:301‐310. 12001991 10.1038/nrc780

[72] Hayden MS , West AP , Ghosh S . NF‐κB and the immune response. Oncogene. 2006;25:6758‐6780. 17072327 10.1038/sj.onc.1209943

[73] Guo Q , Jin YZ , Chen XY , et al. NF‐κB in biology and targeted therapy: new insights and translational implications. Signal Transduct Tar. 2024;9:53. 10.1038/s41392-024-01757-9 PMC10910037 38433280

