# Supplementary figures and images for: Corneal strain influences keratocyte proliferation and migration through upregulation of ALDH3A1 expression
Source: FASEB J. 2024 Dec 9;38(23):e70236. doi: 10.1096/fj.202401392R (PMC11627209; doi:10.1096/fj.202401392R)

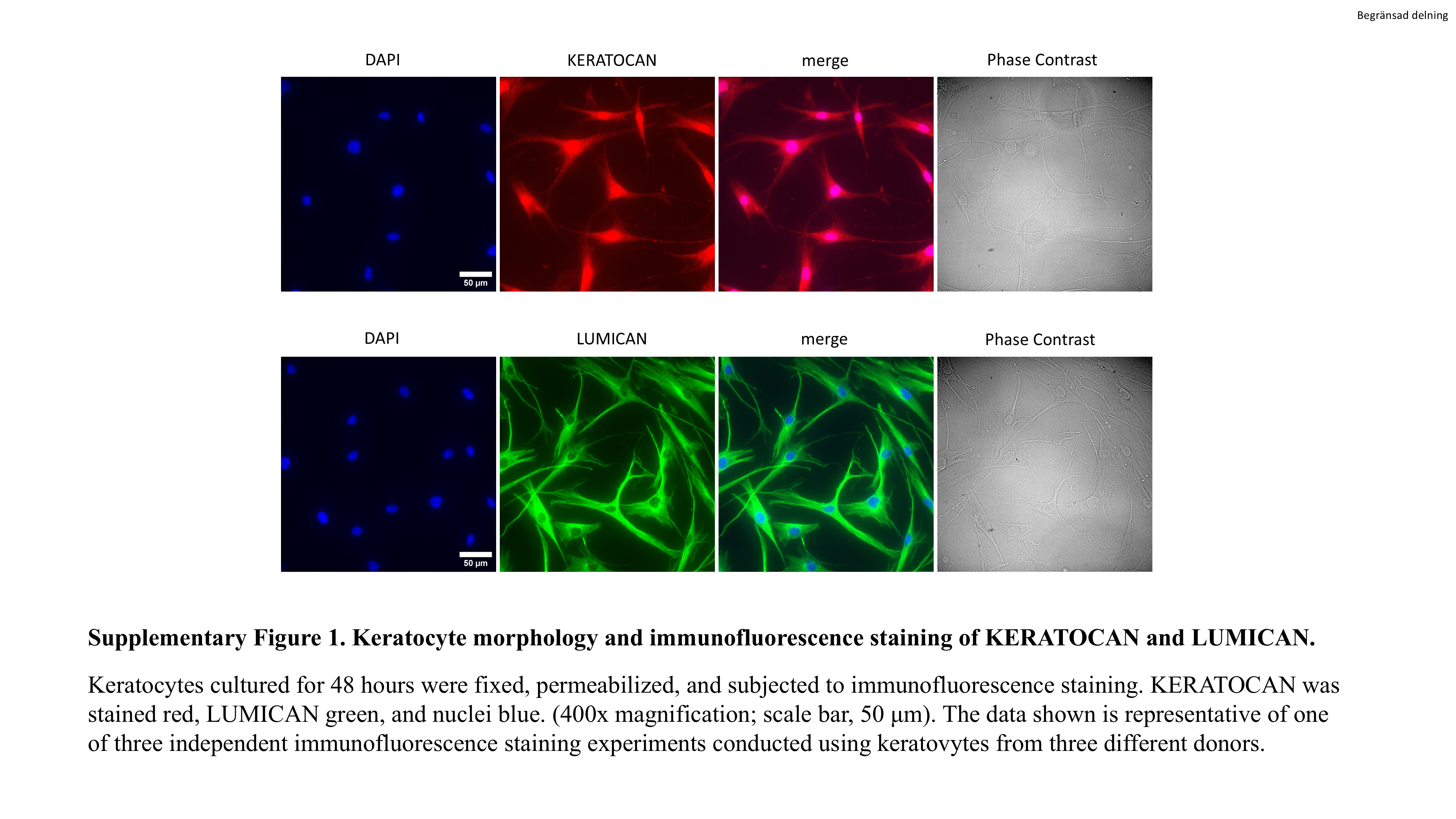

Supplement: Supplementary file 1 — Figure S1. [file FSB2-38-e70236-s001.tif]
