# Supplementary material for: Corneal strain influences keratocyte proliferation and migration through upregulation of ALDH3A1 expression
Source: FASEB J. 2024 Dec 9;38(23):e70236. doi: 10.1096/fj.202401392R (PMC11627209; doi:10.1096/fj.202401392R)
Supplement: Supplementary file 2 — Table S1. [file FSB2-38-e70236-s002.docx]

**Supplementary table 1. Ct values for the keratocyte-specific markers *LUMICAN* and *KERATOCAN***

| **Treatment** | | **Sample ID** | **Ct-value (each row represents an individual donor tested in triplicate)** | | |
| --- | --- | --- | --- | --- | --- |
|  |  |  | ***LUMICAN*** | ***KERATOCAN*** | ***GAPDH*** |
| **Control (untreated**) | 24 h | B280 | 17,172 17,069 17,243 | 29,988 28,653 28,542 | 16,983 16,759 16,755 |
|  |  | B286 | 17,963 17,971 18,665 | 29,660 29,680 30,207 | 15,938 15,979 16,504 |
|  | 48h | B302 | 21,458 21,514 21,545 | 33,184 33,475 33,007 | 18,136 18,154 18,239 |
|  |  | B286 | 16,868 16,757 16,785 | 31,503 31,836 31,701 | 15,910 15,906 15,898 |
|  |  | B315 | 19,813 19,829 19,842 | 27,135 27,135 27,153 | 17,550 17,508 17,555 |
|  |  | B314 | 19,916 19,913 19,962 | 31,468 31,647 31,576 | 16,881 16,854 16,946 |
|  |  | B281 | 18,429 18,449 18,415 | 32,105 32,089 32,014 | 16,977 16,971 17,044 |
|  |  | B309 | 21,915 21,952 21,953 | 31,775 31,882 31,867 | 19,394 19,383 19,496 |
|  | 72h | B288 | 17,300 17,424 17,443 | 31,719 31,688 31,548 | 17,740 17,824 17,954 |
|  |  | B281 | 17,570 17,608 17,213 | 28,185 28,208 27,502 | 17,990 18,100 18,159 |
| **3 % strain** | 24h | B280 | 17,388 17,289 17,238 | 29,131 29,226 29,280 | 16,504 16,337 16,500 |
|  |  | B286 | 18,254 18,095 18,065 | 30,049 30,017 30,068 | 16,129 16,021 16,054 |
|  | 48h | B281 | 18,216 18,224 18,162 | 33,166 34,325 34,314 | 17,123 17,153 17,144 |
|  |  | B286 | 17,272 17,009 17,117 | 32,928 32,122 32,311 | 16,084 15,915 15,864 |
|  | 72h | B281 | 18,052 17,839 17,930 | 28,684 28,906 29,036 | 18,264 18,195 18,070 |
|  |  | B286 | 16,438 16,375 16,396 | 30,135 30,314 30,097 | 16,698 16,666 16,627 |
| **IL-1β treatment** | 24h | B288 | 20,471 20,483 20,397 | 35,477 35,058 35,348 | 17,752 17,730 17,733 |
|  | 48h | B288 | 19,849 19,767 19,841 | 34,723 33,858 34,373 | 17,245 17,320 17,378 |
|  | 72h | B288 | 19,369 19,306 19,383 | 34,109 35,562 34,996 | 17,560 17,661 17,594 |
| **NT siRNA transfection** | 48h | B302 | 20,261 20,217 20,194 | 32,378 32,305 32,330 | 17,106 16,898 16,932 |
|  |  | B315 | 20,126 20,169 20,055 | 26,824 26,802 26,775 | 17,373 17,330 17,226 |
|  |  | B288 | 20,102 20,049 20,068 | 32,252 32,460 32,279 | 17,825 17,548 17,462 |
| **ALDH3A1 siRNA transfection** | 48h | B302 | 20,215 20,211 20,154 | 32,977 33,329 32,973 | 17,016 16,987 16,876 |
|  |  | B315 | 20,154 20,142 20,120 | 27,188 27,140 27,134 | 17,337 17,228 17,194 |
|  |  | B288 | 20,151 20,144 20,094 | 32,791 32,577 33,060 | 17,348 17,379 17,341 |
| **Negative control (****primary human muscle cells)** | 48h | M16M | 38,396 36,952 38,708 | Undetermined | 19,589 19,602 19,683 |
